# Rationale and guidance for strengthening infection prevention and control measures and antimicrobial stewardship programs in Bangladesh: a study protocol

**DOI:** 10.1186/s12913-022-08603-0

**Published:** 2022-10-07

**Authors:** Md. Golam Dostogir Harun, Md Mahabub Ul Anwar, Shariful Amin Sumon, Md. Zakiul Hassan, Tahrima Mohsin Mohona, Aninda Rahman, Syed Abul Hassan Md Abdullah, Md Saiful Islam, S. Cornelia Kaydos-Daniels, Ashley R. Styczynski

**Affiliations:** 1 Programme for Emerging Infections, Infectious Diseases Division, icddr,b, Dhaka, Bangladesh; 2 Centers for Disease Control and Prevention (CDC), Bangladesh Country Office, Dhaka, Bangladesh; 3 Communicable Disease Control, Directorate General of Health Services, Dhaka, Bangladesh; 4 SafetyNet, Dhaka, Bangladesh; 5 University of New South Wales, Sydney, Australia; 6 Division of Infectious Diseases and Geographic Medicine, Stanford University, Stanford, USA

**Keywords:** Infection prevention and control, Hospital-acquired infections, Antimicrobial stewardship, Antimicrobial resistance, Healthcare quality improvement, Tertiary hospital

## Abstract

**Background:**

Hospital-acquired infections (HAIs) and antimicrobial resistance (AMR) are major global health challenges. Drug-resistant infectious diseases continue to rise in developing countries, driven by shortfalls in infection control measures, antibiotic misuse, and scarcity of reliable diagnostics. These escalating global challenges have highlighted the importance of strengthening fundamental infection prevention and control (IPC) measures and implementing effective antimicrobial stewardship programs (ASP). This study aims to present a framework for enhancing IPC measures and ASP efforts to reduce the HAI and AMR burden in Bangladesh.

**Methods:**

This implementation approach will employ a mixed-methods strategy, combining both quantitative and qualitative data from 12 tertiary hospitals in Bangladesh. A baseline assessment will be conducted using the Infection Prevention and Control Assessment Framework (IPCAF) developed by the WHO. We will record IPC practices through direct observations of hand hygiene, personal protective equipment (PPE) utilization, and hospital ward IPC infrastructure. Additionally, data on healthcare providers’ knowledge, attitudes, and practices regarding IPC and antibiotic prescribing will be collected using both structured questionnaires and qualitative interviews. We will also assist the hospital leadership with establishing and/or strengthening IPC and ASP committees. Based on baseline assessments of each healthcare facility, tailored interventions and quality improvement projects will be designed and implemented. An end-line assessment will also be conducted after 12 months of intervention using the same assessment tools. The findings will be compared with the baseline to determine changes in IPC and antibiotic stewardship practices.

**Discussion:**

Comprehensive assessments of healthcare facilities in low-resource settings are crucial for strengthening IPC measures and ASP activities,. This approach to assessing existing IPC and ASP activities will provide policy-relevant data for addressing current shortfalls. Moreover, this framework proposes identifying institutionally-tailored solutions, which will ensure that response activities are appropriately contextualized, aligned with stakeholder priorities, and offer sustainable solutions.

**Conclusion:**

Findings from this study can guide the design and implementation of feasible and sustainable interventions in resource-constrained healthcare settings to address gaps in existing IPC and ASP activities. Therefore, this protocol will be applicable across a broad range of settings to improve IPC and ASP and reduce the burden of hospital-acquired infections and AMR.

**Supplementary Information:**

The online version contains supplementary material available at 10.1186/s12913-022-08603-0.

## Contributions to the literature

- Healthcare facilities in resource-constrained settings require context-specific evidence to identify and respond to gaps in infection control and antibiotic stewardship activities. 
- The study findings can be utilized to guide the development and implementation of effective, sustainable, and tailored interventions to fill gaps in existing infection prevention and control and antimicrobial stewardship programs activities and provide policy-relevant data.
- This protocol provides a guide for a comprehensive assessment of a healthcare setting that can identify multimodal strategies for improving IPC practices and reducing the burden of hospital-acquired infections and antimicrobial resistance.

## Background

Hospital-acquired infections (HAIs) and antimicrobial resistance (AMR) are major global health challenges recognized worldwide [1, 2]. The true global burden of HAIs remains unknown, despite being the most frequent adverse event in health care. Low- and middle-income countries (LMICs) are particularly affected. Although the global overall incidence of HAIs has reportedly fallen over time [1], the pooled prevalence remains significantly higher in resource-constrained settings: 15.5% in LMICs compared to 7.6% in high-income countries [3, 4]. However, this picture of the endemic burden of HAIs in developing countries is extremely fragmented owing to a scarcity of reliable data [4, 5]. Additionally, drug-resistant infections continue to rise in these countries driven by shortfalls in infection control and irrational antibiotic use [6, 7]. HAIs, and especially drug-resistant HAIs, adversely impact patient care and lead to prolonged hospital stays, long-term disability, substantial morbidity and mortality, and significant economic loss [8, 9]. These escalating global challenges have highlighted the importance of fundamental infection prevention and control (IPC) measures and effective antimicrobial stewardship programs (ASP) when providing healthcare, especially during health emergencies such as the COVID-19 pandemic, to ensure patient safety.

Deficits in IPC measures put hospitalized patients in LMICs such as Bangladesh at greater risk of acquiring infections. These include the lack of functional handwashing stations, inadequate bed spacing and isolation units, insufficient equipment decontamination, poor sanitation, improper waste management, and inappropriate use of invasive devices and antibiotics [10, 11]. A study conducted in 2011 in Dhaka Medical College showed that about 30% of hospitalized patients in general surgery and burn wards developed surgical site infections (SSI). This is consistent with another study that documented that the most frequent type of HAI is SSIs (29.1%), followed by urinary tract infections (23.9%), bloodstream infections (19.1%), and healthcare-associated pneumonia (14.8%), including ventilator-associated pneumonia [12]. The majority of hospitalized patients tend to be of advanced age with comorbidities and/or compromised immune systems, making them especially vulnerable to acquiring nosocomial infections [13]. To combat the key challenges of HAIs in tertiary healthcare facilities of Bangladesh, effective IPC programs must be in place.

Antibiotic therapy is regarded as one of the foremost advances in modern medicine that has saved millions of lives since its discovery nearly a century ago [14]. However, the misuse and overuse of antibiotics have contributed significantly to the development of resistant organisms, resulting in an estimated 700,000 deaths annually [15, 16]. If the current trend continues, 10 million deaths annually will be attributed to AMR by 2050, and almost 100 trillion USD will be lost if substantive measures are not taken [17, 18]. The World Health Organization (WHO) antibiotic surveillance report has shown that multidrug-resistant (MDR) organisms and methicillin-resistant Staphylococcus aureus (MRSA) in hospital settings are particularly prevalent in South-East Asian countries [19]. The high proportion of resistant organisms in this region is attributed to sub-optimal hygiene conditions, poor IPC measures, lack of surveillance, and limited ASPs [20, 21]. The function of ASPs is to promote appropriate use of antimicrobials through the implementation of evidence-based, multidisciplinary interventions and is considered an integral component of the health system response to AMR [22, 23]. Studies have shown that antibiotic use, health care expenditure, and nosocomial infections drop significantly following the implementation of ASP without negative impacts on patient outcomes [24, 25]. In Bangladesh, to the best of our knowledge, there are no ASPs in any hospital.

For resource-constrained settings like Bangladesh, significant deficits in IPC lie in the limited availability of essential resources, insufficiently trained personnel, and lack of infection control policies [26]. It is possible to prevent HAIs through the application of a multimodal strategy, if key elements of IPC are adequately followed [26]. There are several IPC guidelines and tools developed by the WHO for assessing IPC practices. Quality Improvement Secretariat (QIS), an initiative under the Directorate General of Health Services (DGHS), Bangladesh, tailored those guidelines and tools for hospital infection control in Bangladesh [27]. According to DGHS,, all tertiary health care facilities are required to have a dedicated team for IPC along with established IPC policies. However, very few IPC programs have been implemented due to a lack of familiarity with IPC and inconsistent monitoring of compliance with this directive. In close collaboration with DGHS, the Director of Communicable Diseases, and the Director of Hospital and Clinics, the goal of this implementation research is to identify strategies to form and strengthen IPC and ASP committees in selected tertiary care hospitals. Involving these IPC and ASP committees in developing targeted interventions using existing resources will make the interventions contextually relevant and sustainable as well as create a sense of ownership to improve the quality and safety of healthcare. The overall aim of this study is to develop and implement a feasible system for improving IPC and ASP measures to reduce the HAI and AMR burden in tertiary care hospitals in Bangladesh.

## Methods

### Study design

This implementation assessment will employ a mixed-methods approach, combining both quantitative and qualitative data to address the study objectives. We will collect the data through observations, in-depth interviews (IDI), focus group discussions (FGD), and structured questionnaires to gather information related to IPC practices and antibiotic use.

### Study setting, population and duration

This project will be piloted in 12 tertiary hospitals (8 government, 2 military, and 2 private) across Bangladesh. The assessment and implementation will be conducted from October 2020 to September 2022. The study population includes IPC and ASP committee members; healthcare providers who are directly involved with patient care such as physicians, nurses, interns; cleaning staff (cleaners, ward boy, non-medical ward staff; i.e. ayas), and patients, including their attendants, of the selected tertiary hospitals.

### Study implementation

To accomplish the project aim, we plan to do the following activities in collaboration with DGHS, hospital leadership, and IPC and ASP committees:

#### Establishing and Strengthening hospital IPC and ASP committees

Before the assessment, we will establish and/or strengthen the IPC and ASP committees in each facility. Initially, we will review the composition of existing IPC and ASP committees in each of the study sites. Subsequently, these committees will be formed and/or modified in collaboration with respective hospital authorities to ensure multidisciplinary representation and adequate qualifications of the committee members (as per ministry of health guidelines and WHO policy guidance about the composition of the committees) [27, 28]. The research team, IPC and ASP committees, and hospital leadership will work closely to enhance the overall IPC and ASP efforts through identifying deficits to be targeted with iterative quality improvement projects.

#### Conducting comprehensive baseline assessment

To understand the existing IPC situation of hospitals, a comprehensive assessment of each healthcare facility will be conducted.Data will also be collected on existing antibiotic use and supply from all study sites to achieve insights surrounding the AMR burden. For this, the project team will conduct a baseline assessment using the following tools:

i. WHO IPCAF facility assessment tool

Firstly, we will assess the IPC level of the healthcare facilities using the WHO Infection Prevention and Control Assessment Framework (IPCAF) questionnaire (Additional file 1: Annexure-I). IPCAF is a diagnostic tool developed in 2018 to support the implementation of the WHO core components of IPC programs at the acute healthcare facility level [29]. It will be used to assess existing IPC activities and resources and identify strengths and gaps. It comprises eight sections reflecting the eight core components and addresses a total of 81 indicators. The IPCAF will categorize hospitals on a continuum of improvement from “inadequate” to “advanced” based on the facility’s total score (out of 800 maximum). The core components are:

- Core component 1: IPC program
- Core component 2: IPC guidelines
- Core component 3: IPC education and training
- Core component 4: Healthcare-associated infection surveillance
- Core component 5: Multimodal strategies
- Core component 6: Monitoring/audits of IPC practices and feedback
- Core component 7: Workload, staffing, and bed occupancy
- Core component 8: Built environment, materials, and equipment for IPC

According to the IPCAF score, the facility IPC status will be categorized as follows:

**Table:** 

SCORE	CATEGORY	INTERPRETATION
0–200	Inadequate	IPC core components implementation is deficient. Significant improvement is required.
201–400	Basic	Some aspects of the IPC core components are in place, but not sufficiently implemented. Further improvement is required.
401–600	Intermediate	Most aspects of IPC core components are appropriately implemented. Continue to improve the scope and quality of implementation and focus on the development of long-term plans to sustain and further promote the existing IPC programme.
601–800	Advanced	The IPC core components are fully implemented according to the WHO recommendations and appropriate to the needs of the facility.

ii. Observation of hand hygiene and personal protective equipment (PPE) use

Secondly, IPC practices among healthcare providers, patients, and visitors will be observed in hospital settings. The assessment of hand hygiene compliance among doctors and nurses will be evaluated against the WHO 5 moments in provider-patient interactions (Additional file 1: Annexure-II). Maintenance of hand hygiene during food and medicine distribution and handling of the patient file will also be observed with standard WHO and QIS observation checklists contextualized for the study setting. Observation sessions will be conducted at various times of the day (i.e. morning shift, evening shift), with different healthcare service providers, and at least 100 hand hygiene opportunities will be observed during each session. Appropriate use of PPE, particularly masks and gloves, will also be noted among healthcare service providers as well as patients and their visitors (Additional file 1: Annexure III and IV).

Data collectors will be coached to observe discretely from a corner of the ward, having limited interaction with either healthcare providers or patients to minimize observer bias. The focus of observation, IPC practices, will also not be disclosed to the study subjects to prevent alteration of their behaviour due to awareness of being observed.

iii. Observation of hospital IPC infrastructure

Thirdly, we will assess hospital infrastructure through direct observation to understand the available facilities for conducting IPC practices. For an effective infection control program, adequate infrastructural support must be in place. Hand hygiene stations will be assessed for the presence of running water, soap, tissues, and signage denoting the essential steps of handwashing. The availability of hand hygiene stations at the point of care will also be noted. Additionally, the cleanliness of the wards, nursing stations, and washrooms will be observed along with management of hospital waste and spills. Data on the number of beds, patients, and attendants in each hospital ward will be collected to further understand the healthcare facilities’ infrastructural sufficiency. The patient-to-bed ratio, healthcare provider-to-patient ratio, and attendants per patient will also be calculated. All the monitoring tools will be adopted from the WHO and QIS and DGHS IPC and ASP manuals (Additional file 1: Annexure V).

iv. Knowledge, attitudes, and practices survey of healthcare service providers concerning IPC

Next, we will assess the knowledge, attitudes, and practices (KAP) of healthcare providers concerning IPC associated factors in healthcare facilities.

All healthcare providers including doctors, nurses, and cleaning staff are eligible to be included in the study and will be selected for the interview through a random sampling technique. A pre-tested semi-structured questionnaire will be used to collect data. Quantitative KAP survey questions (Additional file 1: Annexure VI) are divided into components which include

- Knowledge related to IPC
- Attitudes related to IPC
- Practices related to IPC

We will score 1 point for each correct answer or answer that supports IPC while 0 will be given for incorrect responses or answers that disregard IPC. The percentage of correct responses will be calculated for each participant. Each component of the KAP will subsequently be divided in to three sub-categories based on the following cut-off values of scores: Good (scoring >  = 75%marks), Fair (scoring between 50–75% marks) and Poor (scoring < 50% marks). For each KAP component, good sub-category will be marked as ‘correct knowledge”, ‘favorable attitude’ and ‘safe practice’ respectively.

xxii. Collect antibiotic prescription, supply and consumption information and culture and sensitivity (CS) reports

To assess the rational use of antibiotics, we will collect data on physicians’ antibiotic prescription practices using a self-administered semi-structured questionnaire (Additional file 1: Annexure-VII) developed based on existing literatures and published articles. Information regarding physicians’ awareness AMR, factors that influence their antibiotic prescription practice and their understanding regarding ASP and their functions. We will also collect data on antibiotic supply and consumption from the registrar book of hospital pharmacies, the hospital’s central drug store and patients’ medical records each month.The data will be analyzed using the Global Point Prevalence Survey of Antimicrobial Consumption and Resistance (Global-PPS) tool to measure and monitor antimicrobial prescribing and resistance. The collected data on prescription and consumption of antibiotics will also be quantified according to the WHO AWaRe Classification Database [30] to produce inferences about the overall hospital antibiotic use pattern in tertiary care hospitals of Bangladesh. CS reports (if available) will also be collected from patients and hospital microbiology laboratories to determine the antibiotic resistance patterns in a given facility.

vi. Qualitative assessment with healthcare providers on IPC and AMR

We will conduct a qualitative assessment of healthcare providers to understand how and what IPC and ASP activities can be feasibly implemented or scaled up to reduce HAI and AMR burdens at healthcare facilities. We will conduct key informant interviews (KIIs) as well as separate focus group discussions (FGDs) for physicians and nurses to collect qualitative data on practices, guidelines, system development, and barriers regarding IPC and ASP implementation. We will evaluate the perceptions and understanding of healthcare providers about HAIs, IPC, AMR, vaccine-preventable diseases, and isolation and cohorting, as well as a brief discussion on IPC measures taken during the COVID-19 pandemic. We will compile their recommendations for improving IPC and antibiotic stewardship measures and discuss them with the IPC and ASP committees (Additional file 1: Annexure VIII and IX). Challenges and opportunities for effective infection control and judicious antibiotic use will emerge from these qualitative assessments.

#### Baseline result sharing and intervention design

After completing the baseline assessment and survey, the project team will organize a national level workshop where we will share the baseline information with stakeholders from the Ministry of Health and Family Welfare (MOHFW) and respective hospital authorities. We will also organize similar meetings in each hospital to disseminate the baseline results with the hospital IPC and ASP committees and hospital staff within 3 months of conducting the assessment. Thus, the hospital authorities and committees will be aware of the existing gaps in IPC and ASP activities and be able to recommend context-specific intervention strategies. The findings will hence guide the research team and the respective hospital IPC and ASP committees to collaboratively develop sustainable and feasible interventions. These interventions will be implemented through a tailored approach in collaboration with corresponding hospital authorities.

#### Implementation of tailored interventions

The research team in collaboration with IPC and ASP committees will jointly implement the interventions under hospital leadership. The committees will monitor IPC and antibiotic use practices along with the research team through the use of monitoring checklists and observation tools deployed for the baseline assessment (Additional file 1: Annexure II-V). Systematic collection of data using the tools will enable the committees to establish data-guided initiatives and assess compliance throughout the study period. The project team will assign dedicated physicians and nurses in each study hospital who will assist in implementing the tailored interventions. The IPC and ASP committees will also form IPC monitoring teams, within selected wards of each hospital. The IPC core operational teams, consisting of two IPC committee members, a physician, and anurse from the respective ward, will be responsible for the implementation of IPC measures in daily practices. The ASP monitoring teams, which will include two ASP committee members, a physician, one nurse and a microbiologist from each department will be tasked with executing antimicrobial stewardship activities within designated wards. The hospital IPC monitoring teams and the project team will jointly create awareness regarding infection control through organizing educational programs to improve aspects such as hand hygiene compliance, reduce overcrowding, and enhance environmental cleaning using a quality improvement framework. Based on the local resistance profiles from the CS reports collected by ASP monitoring team, the project team and the ASP committee will develop antibiograms and, with suggestions from senior physicians and microbiologists, produce antibiotic guidelines for selected diseases like diarrhea and urinary tract infections to ensure judicious use of antibiotics. ASP committees will also oversee the compliance of antibiotic use guidelines through random prescription and/or medical chart audits and periodic meetings with physicians to provide feedback on their antibiotic prescribing practices. We will support the teams by providing guidelines and informational materials on IPC and ASP, assisting with modifying observation tools and checklists, conducting training on the quality improvement process, and facilitating logistics for the overall coordination of IPC and ASP activities in each hospital.

#### Monitoring and feedback

The hospital IPC monitoring team will conduct unannounced ward visits and prepare regular activity reports for the IPC and ASP committees based on their findings. Additionally, the project team, in collaboration with DGHS, will arrange a quarterly meeting with the IPC and ASP committees of each healthcare facility to discuss the ongoing IPC activities and troubleshoot any emergent problems. The study investigators will systematically document the process, focusing on the contextual factors and their influence upon the implementation process. Information will be gathered on different approaches to understanding the process, including field-level activities, meetings, negotiations, decisions, planning, and implementation. Documentary materials will include meeting minutes/notes, workshop proceedings and decisions, invitation letters for the implementation partners, field diaries of project staff outlining their observations and experiences as well as images captured during the baseline assessment, unannounced visits, and post-intervention period to establish objective, longitudinal comparisons. Midline assessments will be conducted after 6 months of the intervention in each hospital. The findings of the process documentation and midline assessment will help to monitor the effectiveness of IPC tools and assess the feasibility and acceptability of proposed interventions. Intervention strategies may be modified to overcome any gaps identified as per feedback.

#### Conducting endline assessment

The project team will conduct the endline assessment after 12 months of intervention. We will use similar instruments (Additional file 1: Annexure I-VII) and procedures as doneat baseline to collect data. The entire assessment findings will be systematically documented and compared with the baseline results to identify the changes in IPC and antibiotic use practices at the health facilities throughout implementation. Endline results will be disseminated to the respective hospitals’ IPC and ASP committees and hospital authorities.

#### Assess the functionality of the committees

**Fig.1:**
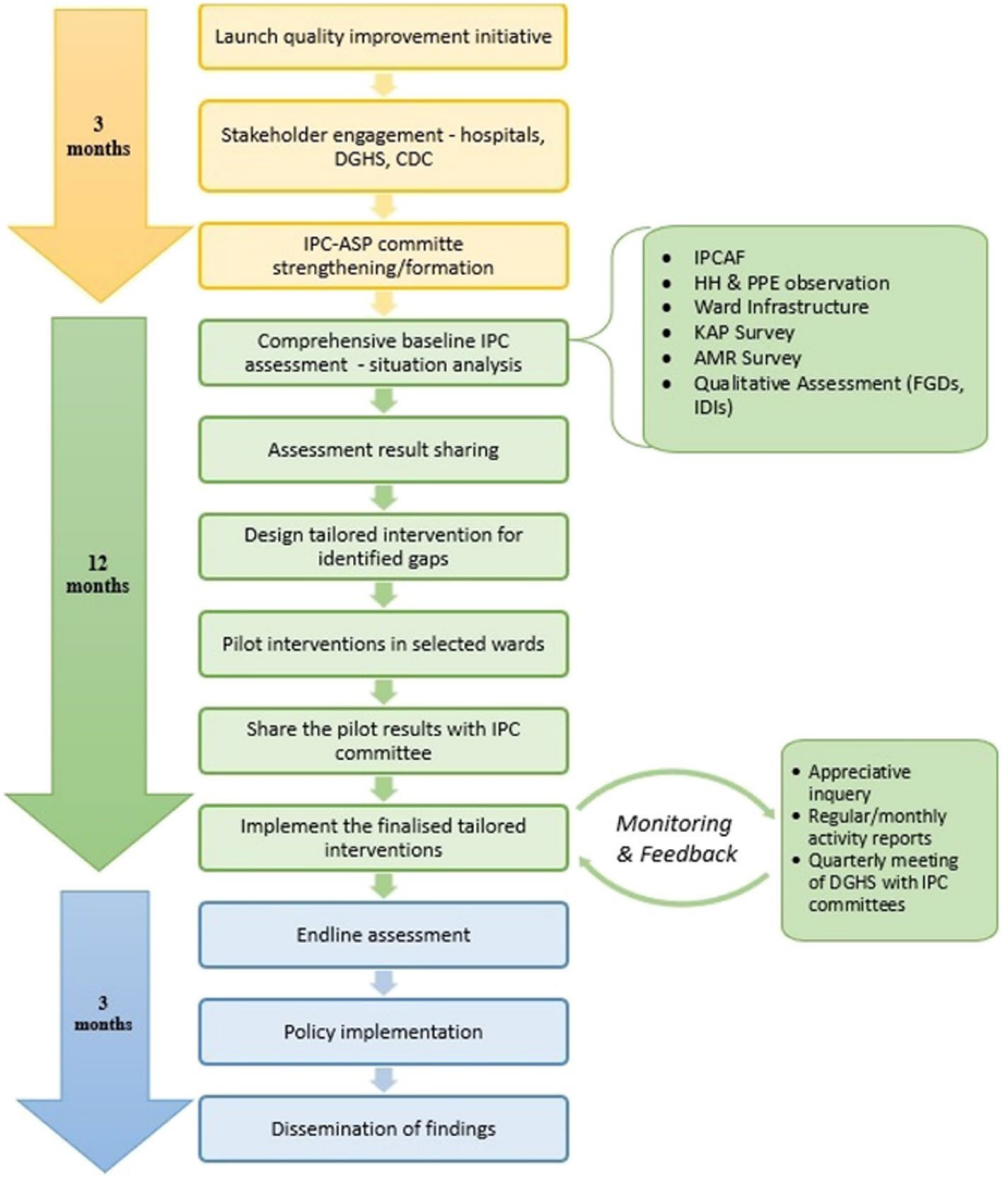
Conceptual framework for improving IPC and ASP measures in healthcare settings

To ensure the sustainability of the proposed interventions after the study period, the research team will measure the functionality of the ASP and IPC committees. The total number of IPC and ASP monitoring teams formed, CS reports collected, local antibiograms developed,meetings and trainings organized, and activity reports submitted in each hospital will be periodically recorded and used as indicators to evaluate the performance of the IPC and ASP committees. The project will be handed over to the IPC and ASP committees and respective hospital authorities at the end of the study period (Fig. 1).

### Outcome (Primary and Secondary) Measures

This study is expected to provide valuable insight on implementation bottlenecks as well as the future scalability of IPC and ASP interventions.

Assessment survey outcome variables include:

- IPCAF score in 12 tertiary healthcare facilities
- % of healthcare providers complying with hand hygiene recommendations
- % of healthcare providers, patients, and attendants using PPE appropriately
- Summary measures of healthcare providers’ knowledge about IPC
- % of healthcare providers practicing appropriate IPC
- Summary of healthcare providers’ attitudes towards IPC
- Patient-to-bed ratio in hospital wards
- Attendants per patient ratio in hospital wards
- Antibiotic use pattern
- Development of antibiogram

The effectiveness of the implementation will be measured and evaluated by the following indicators:

- Number of IPC monitoring teams and ASP monitoring teams formed
- Number of meetings held in the hospitals organized by IPC and ASP committees
- Number of activity reports prepared
- % of healthcare providers receiving basic IPC training
- % of healthcare providers maintaining hand hygiene
- % of physicians using ASP guidelines to prescribe antibiotics

## Statistical analysis

### Sample size calculation

We will calculate the sample size using two independent proportion formulas based on existing estimates of infection prevention knowledge, attitudes, and practices among healthcare providers (71%, 66%, and 43%) and physician awareness of AMR (52%) in Bangladesh [31–33]. We will use a 5% margin of error, 95% confidence interval, 4% effect size for the IPC KAP survey, 8% effect size for physician antibiotic practices survey, and 5% non-response rate.The sample size will be allocated to each selected healthcare facility based on the proportion of healthcare providers relative to the size of each facility. We will use random sampling method to select study participants and a staff list provided from the human resource department of the respective hospitals as a sampling frame. For the qualitative assessment, we will conduct KIIs until saturation of key ideas is reached [34]. Given existing resources, we will conduct 36 FGDs from twelve hospitals, including one FGD on AMR with physicians and one FGD on IPC with physicians and nursing staff from each hospital.

### Data analysis

The analysis of the quantitative data will be based on the assessment of the outcome indicators and a comparison of these indicators between two sequential phases of the study. Outcome indicators will be summarizedusing frequencies, percentages, minimum and maximum statistics,and arithmetic means (standard deviation) or medians (interquartile range), depending on the distribution. The difference-in-differences (DID) estimation technique will be used to examine the change in outcome indicators. Bi-variate and multivariate analysis will also be used to document the changes between baseline and endline surveys after controlling for individual-level demographic and socioeconomic factors.

The qualitative data will be audio-recorded in the native language of participants and then transcribed into English. The qualitative researchers will review the data and develop a code list and definition, which will be shared among team members for consensus.All data will be tabulated in a matrix spreadsheet, which will compress data in an organized way and analyze it by source, code, and theme [35, 36]. The team will then code the findings and categorize the codes under different themes and sub-themes. After that, content analysis will be conducted to identify themes within the data.

## Discussion

A comprehensive assessment of the selected healthcare facilities is the first step towards strengthening IPC and ASP measures by identifying critical gaps. IPC and ASP are highly interrelated and improvements are enhanced by addressing both in parallel. Moreover, enhancing IPC and antibiotic stewardship activities concurrently can lead to a reduction in HAIs and limit the development of AMR [37, 38]. In this protocol paper, we propose to evaluate IPC and ASP practices using existing, validated data collection tools as well as customized observational tools and interview guides for understanding barriers to IPC and antibiotic stewardship. These instruments will be administered to staff at all levels as well as patients and attendants to obtain a wholistic representation. Barriers to IPC and antibiotic stewardship will be iteratively addressed using a QI framework under the direction of IPC and ASP committees.

This will be the first systematic application of IPCAF to conduct IPC healthcare facility assessments in Bangladesh. The usability and reliability of the tool have been validated through global studies [39], and it is well established as an effective diagnostic tool for IPC improvement in healthcare facilities. In Germany, the IPCAF tool was used to assess IPC in 736 hospitals, and 84.5% (622) of facilities were found to be at an advanced level (score > 600) [40]. However, in Pakistan for all 5 hospitals where IPC core components were assessed, the total IPCAF score was less than 200, placing them at an inadequate level [41]. This indicates that IPC implementation may face additional barriers in LMICs and significant improvement is needed. IPC strategies employed by high-income countries may likely not be feasible in LMICs, demonstrating the need for tailored solutions. To strengthen IPC in healthcare facilities in Bangladesh, we must first understand the current IPC situation in healthcare facilities and identify appropriately contextualized solutions for overcoming existing barriers.

Findings from our observational assessments can be used to understand the current infection control practices and develop effective IPC interventions accordingly. At present, many healthcare institutions in LMICSs are under-resourced and overcrowded, with insufficient infrastructure to support effective IPC [42]. Assessment of tertiary healthcare facilities using our monitoring checklist can be used to pinpoint the shortfalls in existing infrastructure and identify areas for prioritization and gain stakeholder support. Current PPE use and hand hygiene practices of healthcare providers should also be assessed as these can contribute to disease spread within hospitals and mayalso represent opportunities for improvement [43]. Hand hygiene compliance with standard alcohol-based hand rub alone among medical personnel was found to have reduced the rate of HAIs by 40% when coupled with appropriate education and sensitization [44, 45]. Similarly, the use of PPE can prevent transmission of infectious diseases, though this can be stymied by supply chain limitations, particularly in the setting of surges in global demand as occurred during the COVID-19 pandemic [46]. Therefore, an assessment of current PPE practices can help ensure appropriate and rational use.

Improving the knowledge and practice of healthcare providers towards infection prevention is paramount to reducing the burden of HAIs. The majority of IPC KAP studies have been conducted among nurses, and results from a systematic review indicated that nurses in most studies had adequate knowledge and positive attitudes but average or poor nursing practices with regards to adherence to IPC standards [47]. A study conducted in northwestern Nigeria revealed nurses were more knowledgeable of the fact that hand hygiene is the most effective method to prevent HAIs and consequently performed better hand hygiene (76%) compared to physicians (52%) [48]. However, another study conducted in 2015 in Pakistan reported doctors to possess better overall knowledge, attitudes, and practices regarding IPC compared to other cadres of healthcare providers [31]. Despite playing a critically important role in IPC, very few studies have been conducted among hospital cleaning staff. The results of one such study conducted in the Kingdom of Saudi Arabia revealed that while the overall knowledge of the hospital staff was relatively good, non-medical staff, in particular, the housekeeping unit, had the lowest level of knowledge about standard IPC measures [49]. Hospital maintenance and cleaning staff are the primary facilitators of environmental cleaning and waste disposal in healthcare settings and yet are often neglected during training programs. No IPC KAP studies of hospital maintenance and cleaning staff have been identified from South Asia. The findings from our KAP survey will provide valuable baseline data for further investigation and necessary interventions to improve the KAP of hospital personnel involved in IPC. The health authorities can then implement specific evidence-based interventions, such as infection control training, which has previously proven to significantly improve IPC practices among physicians, nurses, and cleaning staff in selected hospitals in Bangladesh [50, 51]. Given the increased burden of HAIs in LMICs, there is an urgent need to engage all levels of the healthcare workforce to bridge the gaps in knowledge and practice.

One of the primary goals of ASPs is to ensure judicious use of antibiotics in healthcare settings [52]. Antibiotics are widely used without adherence to standard guidelines in Bangladesh [53, 54]. Various studies in Bangladesh have found polypharmacy and indiscriminate use of antimicrobials to be prevalent among many local physicians, which revealed 81% of prescriptions contained at least two antibiotic drugs [55–57]. Subsequent reports have shown that up to 91% of patients were prescribed antibiotics based on suspicion, without undergoing any cultural-based testing [57, 58]. The prescribing practices of antibiotics by physicians in Bangladesh is cultivating a progressively antibiotic-resistant microbial ecosystem. Findings from our assessments can guide ASP committees in their efforts to assess and improve antibiotic prescribing and consumption practices in hospitals, enhance diagnostic stewardship, and combat the threat of AMR. Subsequently, reports show up to 91% of patients were prescribed antibiotics based on suspicion, without undergoing any cultural-based testing. The prescribing practices of antibiotics by physicians in Bangladesh is cultivating a progressively antibiotic-resistant microbial ecosystem. To slow the emergence of AMR and design antibiotic prescription guidelines, the current antibiotic prescribing practices, supply and usage scenarios, and antibiotic resistance patterns throughout the country need to be understood. Findings from our assessments can guide ASP committees in their efforts to assess and improve antibiotic in hospitals to combat the threat of AMR [59, 60]. An implementation research study conducted in Indonesia over a period of 27 months found a reduction of inappropriate use of antibiotics by about 22% after the implementation of a multifaceted IPC and ASP strategy [61].

Previous interventions to improve IPC have failed to reduce HAI rates in Bangladeshi hospitals as only individual components of IPC have been assessed. Our comprehensive assessment will allow hospitals to prioritize their infection control needs as per identified local barriers and facilitators to infection prevention practices and judicious use of antibiotics. Based on individual needs and baseline assessments of each hospital, tailored interventions can be designed through a participatory approach and implemented in coordination with the IPC and ASP committees within their healthcare institutions. Different hospitals may require different interventions depending on the gaps identified through the comprehensive assessment of each facility. And as both intervention design and implementation will be undertaken in collaboration with hospital authorities using existing resources, it will foster a sense of ownership and improve the sustainability of the interventions.

## Conclusion

A comprehensive assessment of infection control and antibiotic use in tertiary hospitals in Bangladesh and low-resource settings is critically needed to identify and respond to gaps in the existing healthcare system. The results of this research could influence national policies on infection control and antibiotic use in Bangladesh as well as provide a framework for other resource-limited settings.

## Supplementary Information

**Additional file 1.**

## Data Availability

Not applicable.

## Abbreviations

AMR: Antimicrobial Resistance
ASP: Antimicrobial Stewardship Programs
CS: Culture and Sensitivity
DGHS: Directorate General of Health Services
FGD: Focus Group Discussions
HAI: Hospital-acquired Infection
HH: Hand Hygiene
IDI: In-depth Interviews
IPCAF: Infection Prevention and Control Assessment Framework
IPC: Infection Prevention and Control
KAP: Knowledge, Attitudes, and Practices
LMIC: Low-to-middle-income Country
MDRO: Multidrug-Resistant Organism
MOHFW: Ministry of Health and Family Welfare, Bangladesh
PPE: Personal Protective Equipment
QIS: Quality Improvement Secretariat
SSI: Surgical Site Infections
WHO: The World Health Organization
