# Supplementary material for: Rationale and guidance for strengthening infection prevention and control measures and antimicrobial stewardship programs in Bangladesh: a study protocol
Source: BMC Health Serv Res. 2022 Oct 7;22:1239. doi: 10.1186/s12913-022-08603-0 (PMC9540083; doi:10.1186/s12913-022-08603-0)
Supplement: Supplementary file 1 — Additional file 1. [file 12913_2022_8603_MOESM1_ESM.docx]

##
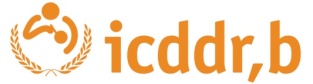
**APPENDIX**

**ANNEXURE-I: FACILITY ASSESSMENT TOOL**

**Infection Prevention and Control Assessment Framework at the Facility Survey**

**Hospital Name:________________________________ID________________________Date:____________**

**Name of the respondent:___________________________Designation:_____________________________**

**Contact no.___________________________**

| **Core component 1: Infection Prevention and Control (IPC) programme** | | |
| --- | --- | --- |
| **Question** | **Answer** | **Score** |
| **1. Do you have an IPC programme?**  Choose one answer | No | 0 |
|  | Yes, without clearly defined objectives | 5 |
|  | Yes, with clearly defined objectives and annual activity plan | 10 |
| **2. Is the IPC programme supported by an IPC team comprising of IPC professionals?** Choose one answer | No | 0 |
|  | Not a team, *only* an IPC focal person | 5 |
|  | Yes | 10 |
| **3. Does the IPC team have at least one full-time IPC professional or equivalent (nurse or doctor working 100% in IPC) available?**  Choose one answer | No IPC professional available | 0 |
|  | No, *only* a part-time IPC professional available | 2.5 |
|  | Yes, one per > 250 beds | 5 |
|  | Yes, one per ≤ 250 beds | 10 |
| **4. Does the IPC team or focal person have dedicated time for IPC activities?** | No | 0 |
|  | Yes | 10 |
| **5. Does the IPC team include both doctors and nurses?** | No | 0 |
|  | Yes | 10 |
| **6. Do you have an IPC committee actively supporting the IPC team?** | No | 0 |
|  | Yes | 10 |
| **7. Are any of the following professional groups represented/ included in the IPC committee?** | | |
| Senior facility leadership (for example, administrative director, chief executive officer [CEO], medical director) | No | 0 |
|  | Yes | 5 |
| Senior clinical staff (for example, physician, nurse) | No | 0 |
|  | Yes | 2.5 |
| Facility management (for example, biosafety, waste, and those tasked with addressing water, sanitation, and hygiene [WASH]) | No | 0 |
|  | Yes | 2.5 |
| **8. Do you have clearly defined IPC objectives (that is, in specific critical areas)?**  Choose one answer | No | 0 |
|  | Yes, IPC objectives *only* | 2.5 |
|  | Yes, IPC objectives and measurable outcome indicators (that is, adequate measures for improvement) | 5 |
|  | Yes, IPC objectives, measurable outcome indicators and set future targets | 10 |
| **9. Does the senior facility leadership show clear commitment and support for the IPC programme:** | | |
| By an allocated budget specifically for the IPC programme (that is, covering IPC activities, including salaries)? | No | 0 |
|  | Yes | 5 |
| By demonstrable support for IPC objectives and indicators within the facility (for example, at executive level meetings, executive rounds, participation in morbidity and mortality meetings)? | No | 0 |
|  | Yes | 5 |
| **10. Does your facility have microbiological laboratory support (either present on or off site) for routine day-to-day use?**  Choose one answer | No | 0 |
|  | Yes, but not delivering results reliably (timely and of sufficient quality) | 5 |
|  | Yes, and delivering results reliably (timely and of sufficient quality) | 10 |
| **Subtotal score** |  | /100 |

| **Core component 2: Infection Prevention and Control (IPC) guidelines** | | |
| --- | --- | --- |
| **Question** | **Answer** | **Score** |
| **1. Does your facility have the expertise (in IPC and/or infectious diseases) for developing or adaptin guidelines?** | No | 0 |
|  | Yes | 7.5 |
| **2. Does your facility have guidelines available for:** | | |
| Standard precautions? | No | 0 |
|  | Yes | 2.5 |
| Hand hygiene? | No | 0 |
|  | Yes | 2.5 |
| Transmission-based precautions?6 | No | 0 |
|  | Yes | 2.5 |
| Outbreak management and preparedness? | No | 0 |
|  | Yes | 2.5 |
| Prevention of surgical site infection?7 | No | 0 |
|  | Yes | 2.5 |
| Prevention of vascular catheter-associated bloodstream infections? | No | 0 |
|  | Yes | 2.5 |
| Prevention of hospital-acquired pneumonia ([HAP]; all types of HAP, including (but not exclusively) ventilator-associated pneumonia)? | No | 0 |
|  | Yes | 2.5 |
| Prevention of catheter-associated urinary tract infections? | No | 0 |
|  | Yes | 2.5 |
| Prevention of transmission of multidrug-resistant (MDR) pathogens? | No | 0 |
|  | Yes | 2.5 |
| Disinfection and sterilization? | No | 0 |
|  | Yes | 2.5 |
| Health care worker protection and safety | No | 0 |
|  | Yes | 2.5 |
| Injection safety? | No | 0 |
|  | Yes | 2.5 |
| Waste management? | No | 0 |
|  | Yes | 2.5 |
| Antibiotic stewardship? | No | 0 |
|  | Yes | 2.5 |
| **3. Are the guidelines in your facility consistent with national/international guidelines (if they exist)?** | No | 0 |
|  | Yes | 10 |
| **4. Is implementation of the guidelines adapted according to the local needs and resources while maintaining key IPC standards?** | No | 0 |
|  | Yes | 10 |
| **5. Are frontline health care workers involved in both planning and executing the implementation of IPC guidelines in addition to IPC personnel?** | No | 0 |
|  | Yes | 10 |
| **6. Are relevant stakeholders (for example, lead doctors and nurses, hospital managers, quality management) involved in the development and adaptation of the IPC guidelines in addition to IPC personnel?** | No | 0 |
|  | Yes | 7.5 |
| **7. Do health care workers receive specific training related to new or updated IPC guidelines introduced in the facility?** | No | 0 |
|  | Yes | 10 |
| **8. Do you regularly monitor the implementation of at least some of the IPC guidelines in your facility?** | No | 0 |
|  | Yes | 10 |
| **Subtotal score** |  | **/100** |

| **Core component 3: Infection Prevention and Control (IPC) education and training** | | |
| --- | --- | --- |
| **Question** | **Answer** | **Score** |
| **1. Are there personnel with IPC expertise (in IPC and/or infectious diseases) to lead IPC training?** | No | 0 |
|  | Yes | 10 |
| **2. Are there additional non-IPC personnel with adequate skills to serve as trainers and mentors (for example, link nurses or doctors, champions)?** Choose one answer | No | 0 |
|  | Yes | 10 |
| **3. How frequently do health care workers receive training regarding IPC in your facility?**  Choose one answer | Never or rarely | 0 |
|  | New employee orientation *only* for healthcare workers | 5 |
|  | New employee orientation and regular (at least annually) IPC training for health care workers  offered but not mandatory | 10 |
|  | New employee orientation and regular (at least annually) mandatory IPC training for all HC workers | 15 |
| **4. How frequently do cleaners and other personnel directly involved in patient care receive training regarding IPC in your facility?**  Choose one answer | Never or rarely | 0 |
|  | New employee orientation *only* for other personnel | 5 |
|  | New employee orientation and regular (at least annually) training for other personnel offered but not mandatory | 10 |
|  | New employee orientation and regular (at least annually) mandatory IPC training for other personnel | 15 |
| **5. Do administrative and managerial staff receive general training regarding IPC in your facility?** Choose one answer | No | 0 |
|  | Yes | 5 |
| **6. How are health care workers and other personnel trained?**  Choose one answer | No training available | 0 |
|  | Using written information and/or oral instruction and/or e-learning *only* | 5 |
|  | Includes *additional* interactive training sessions (for example, simulation and/or bedside training) | 10 |
| **7. Are there periodic evaluations of the effectiveness of training programmes (for example, hand hygiene audits, other checks on knowledge)?** Choose one answer | No | 0 |
|  | Yes, but not regularly | 5 |
|  | Yes, regularly (at least annually) | 10 |
| **8. Is IPC training integrated in the clinical practice and training of other specialties (for example, training of surgeons involves aspects of IPC)?** Choose one answer | No | 0 |
|  | Yes, in some disciplines | 5 |
|  | Yes, in all disciplines | 10 |
| **9. Is there specific IPC training for patients or family members to minimize the potential for health care associated infections (for example, immune suppressed patients, patients with invasive devices, patients with multidrug-resistant infections)?** | No | 0 |
|  | Yes | 5 |
| **10. Is ongoing development/education offered for IPC staff (for example, by regularly attending conferences, courses)?** | No | 0 |
|  | Yes | 10 |
| **Subtotal score** |  | **/100** |

| **Core component 4: Health care-associated infection (HAI) surveillance** | | |
| --- | --- | --- |
| **Question** | **Answer** | **Score** |
| **Organization of surveillance** | | |
| **1. Is surveillance a defined component of your IPC programme?** | No | 0 |
|  | Yes | 5 |
| **2. Do you have personnel responsible for surveillance activities?** | No | 0 |
|  | Yes | 5 |
| **3. Have the professionals responsible for surveillance activities been trained in basic epidemiology, surveillance and IPC (I,e, capacity to oversee surveillance methods, data management, interpretation)?** | No | 0 |
|  | Yes | 5 |
| **4. Do you have informatics/IT support to conduct surveillance (for example, equipment, mobile technologies, electronic health records)?** | No | 0 |
|  | Yes | 5 |
| **Priorities for surveillance - defined according to the scope of care** | | |
| **5. Do you go through a prioritization exercise to determine the HAIs to be targeted for surveillance according to the local context (that is, identifying infections that are major causes of morbidity and mortality in the facility)?** | No | 0 |
|  | Yes | 5 |
| **6. In your facility is surveillance conducted for:** | | |
| Surgical site infections? | No | 0 |
|  | Yes | 2.5 |
| Device-associated infections (for example, catheter associated urinary tract infections, central line associated bloodstream infections, peripheral-line associated bloodstream infections, ventilator associated pneumonia)? | No | 0 |
|  | Yes | 2.5 |
| Clinically-defined infections (for example, definitions based only on clinical signs or symptoms in the absence of microbiological testing)? | No | 0 |
|  | Yes | 2.5 |
| Colonization or infections caused by multidrug  resistant pathogens according to your local epidemiological situation? | No | 0 |
|  | Yes | 2.5 |
| Local priority epidemic-prone infections (for example, norovirus, influenza, tuberculosis [TB], severe acute respiratory syndrome [SARS], Ebola, Lassa fever)? | No | 0 |
|  | Yes | 2.5 |
| Infections in vulnerable populations (for example, neonates, intensive care unit, immunocompromised, burn patients)? | No | 0 |
|  | Yes | 2.5 |
| Infections that may affect health care workers in clinical, laboratory, or other settings (for example, hepatitis B or C, human immunodeficiency virus [HIV], influenza)? | No | 0 |
|  | Yes | 2.5 |
| **7. Do you regularly evaluate if your surveillance is in line with the current needs and priorities of your facility?** | No | 0 |
|  | Yes | 5 |
| **Methods of surveillance** | | |
| **8. Do you use reliable surveillance case definitions (defined numerator and denominator according to international definitions [e.g. CDC NHSN/ECDC]or if adapted, through an evidence-based adaptation process and expert consultation?** | No | 0 |
|  | Yes | 5 |
| **9. Do you use standardized data collection methods (for example, active prospective surveillance) according to international surveillance protocols (for example, CDC NHSN/ECDC) or if adapted, through an evidence based adaptation process and expert consultation?** | No | 0 |
|  | Yes | 5 |
| **10. Do you have processes in place to regularly review data quality (for example, assessment of case report forms, review of microbiology results, denominator determination, etc.)?** | No | 0 |
|  | Yes | 5 |
| **11. Do you have adequate microbiology and laboratory capacity to support surveillance?** Choose one answer | No | 0 |
|  | Yes, can differentiate gram positive/negative strains but cannot identify pathogens | 2.5 |
|  | Yes, can reliably identify pathogens (for example, isolate identification) in a timely manner | 5 |
|  | Yes, can reliably identify pathogens and antimicrobial drug resistance patterns (that is, susceptibilities) in a timely manner | 10 |
| **Information analysis and dissemination/data use, linkage, and governance** | | |
| **12. Are surveillance data used to make tailored unit/facility-based plans for the improvement of IPC practices?** | No | 0 |
|  | Yes | 5 |
| **13. Do you analyze antimicrobial drug resistance on a regular basis (for example, quarterly/half yearly/annually)?** | No | 0 |
|  | Yes | 5 |
| **14. Do you regularly (for example, quarterly/half-yearly/annually) feedback up-to-date surveillance information to:** | | |
| Frontline health care workers (doctors/nurses)? | No | 0 |
|  | Yes | 2.5 |
| Clinical leaders/heads of department | No | 0 |
|  | Yes | 2.5 |
| IPC committee | No | 0 |
|  | Yes | 2.5 |
| Non-clinical management/administration (chief executive officer/chief financial officer)? | No | 0 |
|  | Yes | 2.5 |
| **15. How do you feedback up-to-date surveillance information? (at least annually)**  Choose one answer | No feedback | 0 |
|  | By written/oral information *only* | 2.5 |
|  | By presentation and interactive problem-orientated solution finding | 7.5 |
| **Subtotal score** |  | /100 |

| **Core component 5: Multimodal strategies for implementation of infection prevention and control (IPC) interventions** | | |
| --- | --- | --- |
| **Question** | **Answer** | **Score** |
| **1. Do you use multimodal strategies to implement IPC interventions?** | No | 0 |
|  | Yes | 15 |
| **2. Do your multimodal strategies include any or all of the following elements:**  Choose one answer (the most accurate) per element | **System change** | 0 |
|  | Element not included in multimodal strategies | 0 |
|  | Interventions to ensure the necessary infrastructure and continuous availability of supplies are in place | 5 |
|  | Interventions to ensure the necessary infrastructure and continuous availability of supplies are in place and addressing ergonomics17 and accessibility, such as the best placement of central venous catheter set and tray | 10 |
|  | **Education and training** |  |
|  | Element not included in multimodal strategies | 0 |
|  | Written information and/or oral instruction and/or e-learning *only* | 5 |
|  | *Additional* interactive training sessions (includes simulation and/or bedside training) | 10 |
|  | **Monitoring and feedback** |  |
|  | Element not included in multimodal strategies | 0 |
|  | Monitoring compliance with process or outcome indicators (for example, audits of hand hygiene or catheter practices) | 5 |
|  | Monitoring compliance and providing timely feedback of monitoring results to health care workers and key players | 10 |
|  | **Communications and reminders** |  |
|  | Element not included in multimodal strategies | 0 |
|  | Reminders, posters, or other advocacy/awareness-raising tools to promote the intervention | 5 |
|  | *Additional* methods/initiatives to improve team communication across units and disciplines (for example, by establishing regular case conferences and feedback rounds) | 10 |
|  | **Safety climate and culture change** |  |
|  | Element not included in multimodal strategies | 0 |
|  | Managers/leaders show visible support and act as champions and role models, promoting an adaptive approach18 and strengthening a culture that supports IPC, patient safety and quality | 5 |
|  | *Additionally*, teams and individuals are empowered so that they perceive ownership of the intervention (for example, by participatory feedback rounds) | 10 |
| **3. Is a multidisciplinary team used to implement IPC multimodal strategies?** | No | 0 |
|  | Yes | 15 |
| **4. Do you regularly link to colleagues from quality improvement and patient safety to develop and promote IPC multimodal strategies?** | No | 0 |
|  | Yes | 10 |
| **5. Do these strategies include bundlesor checklists?** | No | 0 |
|  | Yes | 10 |
| **Subtotal score** |  | /100 |
| **Core component 6: Monitoring/audit of IPC practices and feedback** | | |
| **Question** | **Answer** | **Score** |
| **1. Do you have trained personnel responsible for monitoring/audit of IPC practices and feedback?** | No | 0 |
|  | Yes | 10 |
| **2. Do you have a well-defined monitoring plan with clear goals, targets and activities (including tools to collect data in a systematic way)?** | No | 0 |
|  | Yes | 7.5 |
| **3. Which processes and indicators do you monitor in your facility?**  Tick all that apply | None | 0 |
|  | Hand hygiene compliance (using the WHO hand hygiene observation tool20 or equivalent) | 5 |
|  | Intravascular catheter insertion and/or care | 5 |
|  | Wound dressing change | 5 |
|  | Transmission-based precautions and isolation to prevent the spread of multidrug resistant organisms (MDRO) | 5 |
|  | Cleaning of the ward environment | 5 |
|  | Disinfection and sterilization of medical equipment/instruments | 5 |
|  | Consumption/usage of alcohol based handrub or soap | 5 |
|  | Consumption/usage of antimicrobial agents | 5 |
|  | Waste management | 5 |
| **4. How frequently is the *WHO Hand Hygiene Self Assessment Framework Survey*undertaken?**  Choose one answer | Never | 0 |
|  | Periodically, but no regular schedule | 2.5 |
|  | At least annually | 5 |
| **5. Do you feedback auditing reports (for example, feedback on hand hygiene compliance data or other processes) on the state of the IPC activities/performance?**  Tick all that apply | No reporting | 0 |
|  | Yes, within the IPC team | 2.5 |
|  | Yes, to department leaders and managers in the areas being audited | 2.5 |
|  | Yes, to frontline health care workers | 2.5 |
|  | Yes, to the IPC committee or quality of care committees or equivalent | 2.5 |
|  | Yes, to hospital management and senior administration | 2.5 |
| **6. Is the reporting of monitoring data undertaken regularly (at least annually)?** | No | 0 |
|  | Yes | 10 |
| **7. Are monitoring and feedback of IPC processes and indicators performed in a “blame-free” institutional culture aimed at improvement and behavioral change?** | No | 0 |
|  | Yes | 5 |
| **8. Do you assess safety cultural factors in your facility (for example, by using other surveys such as HSOPSC, SAQ, PSCHO, HSC)** | No | 0 |
|  | Yes | 5 |
| **Subtotal score** |  | /100 |

| **Core component 7: Workload, staffing and bed occupancy** | | |
| --- | --- | --- |
| **Question** | **Answer** | **Score** |
| **Staffing** | | |
| **1. Are appropriate staffing levels assessed in your facility according to patient workload using national standards or a standard staffing needs assessment tool such as the *WHO Workload indicators of staffing need* method?** | No | 0 |
|  | Yes | 5 |
| **2. Is an agreed (that is, WHO or national) ratio of health care workers to patients maintained across your facility?**  Choose one answer | No | 0 |
|  | Yes, for staff in less than 50% of units | 5 |
|  | Yes, for staff in more than 50% of units | 10 |
|  | Yes, for all health care workers in the facility | 15 |
| **3. Is a system in place in your facility to act on the results of the staffing needs assessments when staffing levels are deemed to be too low?** | No | 0 |
|  | Yes | 10 |
| **Bed occupancy** | | |
| **4. Is the design of wards in your facility in accordance with international standards regarding bed capacity?**  Choose one answer | No | 0 |
|  | Yes, but *only* in certain departments | 5 |
|  | Yes, for all departments (including emergency department and pediatrics) | 15 |
| **5. Is bed occupancy in your facility kept to one patient per bed?**  Choose one answer | No | 0 |
|  | Yes, but *only* in certain departments | 5 |
|  | Yes, for all units (including emergency departments and pediatrics) | 15 |
| **6. Are patients in your facility placed in beds standing in the corridor outside of the room (including beds in the emergency department)?**  Choose one answer | Yes, more frequently than twice a week | 0 |
|  | Yes, less frequently than twice a week | 5 |
|  | No | 15 |
| **7. Is adequate spacing of > 1 meter between patient beds ensured in your facility?**  Choose one answer | No | 0 |
|  | Yes, but *only* in certain departments | 5 |
|  | Yes, for all departments (including emergency department and pediatrics) | 15 |
| **8. Is a system in place in your facility to assess and respond when adequate bed capacity is exceeded?**  Choose one answer | No | 0 |
|  | Yes, this is the responsibility of the head of department | 5 |
|  | Yes, this is the responsibility of the hospital administration/ management | 10 |
| **Subtotal score** |  | /100 |

| **Core component 8: Built environment, materials and equipment for IPC at the facility level** | | | |
| --- | --- | --- | --- |
| **Question** | **Answer** | | **Score** |
| **Water** | | | |
| **1. Are water services available at all times and of sufficient quantity for all uses (for example, hand washing, drinking, personal hygiene, medical activities, sterilization, decontamination, cleaning and laundry)?**  Choose one answer | No, available on average < 5 days per week | | 0 |
|  | Yes, available on average ≥ 5 days per week or every day but not of sufficient quantity | | 2.5 |
|  | Yes, every day and of sufficient quantity | | 7.5 |
| **2. Is a reliable safe drinking water station present and accessible for staff, patients and families at all times and in all locations/wards?**  Choose one answer | No, not available | | 0 |
|  | Sometimes, or only in some places or not available for all users | | 2.5 |
|  | Yes, accessible at all times and for all wards/groups | | 7.5 |
| **Hand hygiene and sanitation facilities** | | | |
| **3. Are functioning hand hygiene stations (that is, alcohol-based hand rub solution or soap and water and clean single-use towels) available at all points of care?**  Choose one answer | No, not present | | 0 |
|  | Yes, stations present, but supplies are not reliably available | | 2.5 |
|  | Yes, with reliably available supplies | | 7.5 |
| **4. In your facility, are ≥ 4 toilets or improved latrines available for outpatient settings or ≥ 1 per 20 users for inpatient settings?**  Choose one answer | Less than required number of toilets or latrines available and functioning | | 0 |
|  | Sufficient number present but not all functioning | | 2.5 |
|  | Sufficient number present and functioning | | 7.5 |
| **Power supply, ventilation and cleaning** | | | |
| **5. In your health care facility, is sufficient energy/power supply available at day and night for all uses (for example, pumping and boiling water, sterilization and decontamination, incineration or alternative treatment technologies, electronic medical devices, general lighting of areas where health care procedures are performed to ensure safe provision of health care and lighting of toilet facilities and showers)?** Choose one answer | No | | 0 |
|  | Yes, sometimes or only in some of the mentioned areas | | 2.5 |
|  | Yes, always and in all mentioned areas | | 5 |
| **6. Is functioning environmental ventilation (natural or mechanical) available in patient care areas?** | No | | 0 |
|  | Yes | | 5 |
| **7. For floors and horizontal work surfaces, is there an accessible Record of cleaning, signed by the cleaners each day?**  Choose one answer | No record of floors and surfaces being cleaned | | 0 |
|  | Record exists, but is not completed and signed daily or is outdated | | 2.5 |
|  | Yes, record completed and signed daily | | 5 |
| **8. Are appropriate and well-maintained materials for cleaning (for example, detergent, mops, buckets, etc.) available?** Choose one answer | No materials available | | 0 |
|  | Yes, available but not well maintained | | 2.5 |
|  | Yes, available and well maintained | | 5 |
| **Patient placement and personal protective equipment (PPE) in health care settings** | | | |
| **9. Do you have single patient rooms or rooms for cohorting patients with similar pathogens if the number of isolation rooms is insufficient (for example, TB, measles, cholera, Ebola, SARS)?**  Choose one answer | No | | 0 |
|  | No single rooms but rather rooms suitable for patient cohorting available | | 2.5 |
|  | Yes, single rooms are available | | 7.5 |
| **10. Is PPE available at all times and in sufficient quantity for all uses for all health care workers?**  Choose one answer | No | | 0 |
|  | Yes, but not continuously available in sufficient quantities | | 2.5 |
|  | Yes, continuously available in sufficient quantities | | 7.5 |
| **Medical waste management and sewage** | | | |
| **11. Do you have functional waste collection containers for non-infectious (general) waste, infectious waste and, sharps waste in close proximity to all waste generation points?**  Choose one answer | No bins or separate sharps disposal | | 0 |
|  | Separate bins present but lids missing or more than 3/4 full; only two bins (instead of three); or bins at some but not all waste generation points | | 2.5 |
|  | Yes | | 5 |
| **12. Is a functional burial pit/fenced waste dump or municipal pick-up available for disposal of non infectious (non-hazardous/ general waste)?**  Choose one answer | No pit or other disposal method used | | 0 |
|  | Pit in facility but insufficient dimensions; pits/dumps overfilled or not fenced/locked; or irregular municipal waste pick up | | 2.5 |
|  | Yes | | 5 |
| **13. Is an incinerator or alternative treatment technology for the treatment of infectious and sharp waste (for example, an autoclave) present (either present on or off site and operated by a licensed waste management service), functional and of a sufficient capacity?**  Choose one answer | No, none present | | 0 |
|  | Present, but not functional | | 1 |
|  | Yes | | 5 |
| **14. Is a wastewater treatment system (for example, septic tank followed by drainage pit) present (either on or off site) and functioning reliably?** Choose one answer | No, not present | | 0 |
|  | Yes, but not functioning reliably | | 2.5 |
|  | Yes and functioning reliably | | 5 |
| **Decontamination and sterilization** | | | |
| **15. Does your health care facility provide a dedicated decontamination area and/or sterile supply department (either present on or off site and operated by a licensed decontamination management service) for the decontamination and sterilization of medical devices and other items/equipment?** Choose one answer | No, not present | | 0 |
|  | Yes, but not functioning reliably | | 2.5 |
|  | Yes and functioning reliably | | 5 |
| **16. Do you reliably have sterile and disinfected equipment ready for use?**  Choose one answer | No, available on average < five days per week | | 0 |
|  | Yes, available on average ≥ five days per week or every day, but not of sufficient quantity | | 2.5 |
|  | Yes, available every day and of sufficient quantity | | 5 |
| **17. Are disposable items available when necessary? (for example, injection safety devices, examination gloves)**  Choose one answer | No, not available | | 0 |
|  | Yes, but *only* sometimes available | | 2.5 |
|  | Yes, continuously available | | 5 |
| **Subtotal score** |  | | /100 |
| **Interpretation: A three-step process** | | | |
| **1. Add up your points** | | | |
|  | | **Score** | |
| **Section (Core component)** | | **Subtotals** | |
| 1. IPC programme | |  | |
| 2. IPC guidelines | |  | |
| 3. IPC education and training | |  | |
| 4. HAI surveillance | |  | |
| 5. Multimodal strategies | |  | |
| 6. Monitoring/audits of IPC practices and feedback | |  | |
| 7. Workload, staffing and bed occupancy | |  | |
| 8. Built environment, materials and equipment for IPC at the facility level | |  | |
| **Final total score** | | /800 | |
| **2. Determine the assigned “IPC level” in your facility using the total score from Step 1** | | | |
| **Total score** (range) | | **IPC level** | |
| 0–200 | | Inadequate | |
| 201–400 | | Basic | |
| 401–600 | | Intermediate | |
| 601–800 | | Advanced | |

**Source: World Health Organization. 2020, https://www.who.int/infection-prevention/tools/core-components/en/*

**
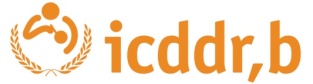
ANNEXURE-II: HAND HYGIENE OBSERVATION FORM**

Data ID:_____ ___Hospital Name : __________ Department : _________ Date: _____/_____/2021

Start time: _____________End time__________ Observed by ________________________

| **Prof Cat.** | **Doctor** | | **Doctor** | | **Nurse** | | **Nurse** | | **Nurse** | | **Cleaner** | |
| --- | --- | --- | --- | --- | --- | --- | --- | --- | --- | --- | --- | --- |
|  | **M / F** | | **M / F** | | **M / F** | | **M / F** | | **M / F** | | **M / F** | |
| **Opp** | **Indication** | **HH Action** | **Indication** | **HH Action** | **Indication** | **HH Action** | **Indication** | **HH Action** | **Indication** | **HH Action** | **Indication** | **HH Action** |
| **1** | B Pt  B Asp  A Fl  A Pt  A Sur  A Pt fl | HR  HW  missed  🌕 gloves | B Pt  B Asp  A Fl  A Pt  A Sur  A Pt fl | HR  HW  missed  🌕 gloves | B Pt  B Asp  A Fl  A Pt  A Sur  A Pt fl | HR  HW  missed  🌕 gloves | B Pt  B Asp  A Fl  A Pt  A Sur  A Pt fl | HR  HW  missed  🌕 gloves | B Pt  B Asp  A Fl  A Pt  A Sur  A Pt fl | HR  HW  missed  🌕 gloves | B Pt  B Asp  A Fl  A Pt  A Sur  A Pt fl | HR  HW  missed  🌕 gloves |
|  |  |  |  |  |  |  |  |  |  |  |  |  |
| **2** | B Pt  B Asp  A Fl  A Pt  A Sur  A Pt fl | HR  HW  missed  🌕 gloves | B Pt  B Asp  A Fl  A Pt  A Sur  A Pt fl | HR  HW  missed  🌕 gloves | B Pt  B Asp  A Fl  A Pt  A Sur  A Pt fl | HR  HW  missed  🌕 gloves | B Pt  B Asp  A Fl  A Pt  A Sur  A Pt fl | HR  HW  missed  🌕 gloves | B Pt  B Asp  A Fl  A Pt  A Sur  A Pt fl | HR  HW  missed  🌕 gloves | B Pt  B Asp  A Fl  A Pt  A Sur  A Pt fl | HR  HW  missed  🌕 gloves |
|  |  |  |  |  |  |  |  |  |  |  |  |  |
| **3** | B Pt  B Asp  A Fl  A Pt  A Sur  A Pt fl | HR  HW  missed  🌕 gloves | B Pt  B Asp  A Fl  A Pt  A Sur  A Pt fl | HR  HW  missed  🌕 gloves | B Pt  B Asp  A Fl  A Pt  A Sur  A Pt fl | HR  HW  missed  🌕 gloves | B Pt  B Asp  A Fl  A Pt  A Sur  A Pt fl | HR  HW  missed  🌕 gloves | B Pt  B Asp  A Fl  A Pt  A Sur  A Pt fl | HR  HW  missed  🌕 gloves | B Pt  B Asp  A Fl  A Pt  A Sur  A Pt fl | HR  HW  missed  🌕 gloves |
|  |  |  |  |  |  |  |  |  |  |  |  |  |
| **4** | B Pt  B Asp  A Fl  A Pt  A Sur  A Pt fl | HR  HW  missed  🌕 gloves | B Pt  B Asp  A Fl  A Pt  A Sur  A Pt fl | HR  HW  missed  🌕 gloves | B Pt  B Asp  A Fl  A Pt  A Sur  A Pt fl | HR  HW  missed  🌕 gloves | B Pt  B Asp  A Fl  A Pt  A Sur  A Pt fl | HR  HW  missed  🌕 gloves | B Pt  B Asp  A Fl  A Pt  A Sur  A Pt fl | HR  HW  missed  🌕 gloves | B Pt  B Asp  A Fl  A Pt  A Sur  A Pt fl | HR  HW  missed  🌕 gloves |
|  |  |  |  |  |  |  |  |  |  |  |  |  |
| **5** | B Pt  B Asp  A Fl  A Pt  A Sur  A Pt fl | HR  HW  missed  🌕 gloves | B Pt  B Asp  A Fl  A Pt  A Sur  A Pt fl | HR  HW  missed  🌕 gloves | B Pt  B Asp  A Fl  A Pt  A Sur  A Pt fl | HR  HW  missed  🌕 gloves | B Pt  B Asp  A Fl  A Pt  A Sur  A Pt fl | HR  HW  missed  🌕 gloves | B Pt  B Asp  A Fl  A Pt  A Sur  A Pt fl | HR  HW  missed  🌕 gloves | B Pt  B Asp  A Fl  A Pt  A Sur  A Pt fl | HR  HW  missed  🌕 gloves |
|  |  |  |  |  |  |  |  |  |  |  |  |  |
| **6** | B Pt  B Asp  A Fl  A Pt  A Sur  A Pt fl | HR  HW  missed  🌕 gloves | B Pt  B Asp  A Fl  A Pt  A Sur  A Pt fl | HR  HW  missed  🌕 gloves | B Pt  B Asp  A Fl  A Pt  A Sur  A Pt fl | HR  HW  missed  🌕 gloves | B Pt  B Asp  A Fl  A Pt  A Sur  A Pt fl | HR  HW  missed  🌕 gloves | B Pt  B Asp  A Fl  A Pt  A Sur  A Pt fl | HR  HW  missed  🌕 gloves | B Pt  B Asp  A Fl  A Pt  A Sur  A Pt fl | HR  HW  missed  🌕 gloves |
|  |  |  |  |  |  |  |  |  |  |  |  |  |
| **7** | B Pt  B Asp  A Fl  A Pt  A Sur  A Pt fl | HR  HW  missed  🌕 gloves | B Pt  B Asp  A Fl  A Pt  A Sur  A Pt fl | HR  HW  missed  🌕 gloves | B Pt  B Asp  A Fl  A Pt  A Sur  A Pt fl | HR  HW  missed  🌕 gloves | B Pt  B Asp  A Fl  A Pt  A Sur  A Pt fl | HR  HW  missed  🌕 gloves | B Pt  B Asp  A Fl  A Pt  A Sur  A Pt fl | HR  HW  missed  🌕 gloves | B Pt  B Asp  A Fl  A Pt  A Sur  A Pt fl | HR  HW  missed  🌕 gloves |
|  |  |  |  |  |  |  |  |  |  |  |  |  |
| **8** | B Pt  B Asp  A Fl  A Pt  A Sur  A Pt fl | HR  HW  missed  🌕 gloves | B Pt  B Asp  A Fl  A Pt  A Sur  A Pt fl | HR  HW  missed  🌕 gloves | B Pt  B Asp  A Fl  A Pt  A Sur  A Pt fl | HR  HW  missed  🌕 gloves | B Pt  B Asp  A Fl  A Pt  A Sur  A Pt fl | HR  HW  missed  🌕 gloves | B Pt  B Asp  A Fl  A Pt  A Sur  A Pt fl | HR  HW  missed  🌕 gloves | B Pt  B Asp  A Fl  A Pt  A Sur  A Pt fl | HR  HW  missed  🌕 gloves |
|  |  | |  | |  | |  | |  | |  | |
|  | TOTAL DOC =_____ | | | | TOTAL NUR =_____ | | | | | | TOTAL CLN =_____ | |

General Instruction:

1. The health-care workers, directly involved in patient care, belonging to one of the main three following professional categories (see below), is observed during the delivery of health-care activities to patients
2. Each column of the grid to record hand hygiene practices is intended to be dedicated to a specific professional category. Therefore, numerous health-care workers may be sequentially included during one session in the column dedicated to their category. Total number of health-care workers from specific professional category observed must be recorded.
3. Each opportunity refers to one line in each column; each line is independent from one column to another.
4. As soon as an indication for hand hygiene is detected, count an opportunity in the appropriate column and tick (√) the square corresponding to the indication(s) detected. Then complete all the indications that apply, and the related hand hygiene actions observed or missed.
5. The observer should record only actions that he or she can clearly see and that correspond to indications; the observer is not allowed to assume that an action has taken place
6. When several indications fall in one opportunity, each one must be recorded by ticking (√) the squares.
7. Performed or missed actions must always be registered within the context of an opportunity.
8. Glove use should be recorded for all health-care worker.

Description:

| **Hospital Name:** | to complete according to the local nomenclature | | |
| --- | --- | --- | --- |
| **Department:** | medical, including dermatology, neurology, haematology, oncology, etc. | | surgery, including neurosurgery, urology, EENT, ophthalmology, etc. |
|  | mixed (medical & surgical), including gynaecology | | obstetrics, including related surgery |
|  | paediatrics, including related surgery | | intensive care & resuscitation |
|  | emergency unit | | long term care & rehabilitation |
|  | ambulatory care, including related surgery | | other (to specify) |
| **Ward/Unit:** | to complete according to the local nomenclature | | |
| **Date:** | day (dd) / month (mm) / year (yy) | | |
| **Start/end time:** | hour (hh) / minute (mm). | | |
| **Observed by:** | observer’s full name (the observer is responsible for the data collection and for checking their accuracy before submitting the form for analysis. | | |
| **Prof.cat:** | according to the following classification: | | |
|  | **1. Doctor** | All type of medical doctors directly involved in patient care including medical interns | |
|  | **2. Nurse** | Nurses, midwives, nursing and midwifery student directly involved in patient care | |
|  | **3. Cleaners** | Support staff such as aya, ward boy, cleaners, etc directly involved with patient care | |
| **Gender of HCW** | Male (M) or female (F) | | |
| **Opp(ortunity):** | defined by one indication at least | | |
| **Indication:** | reason(s) that motivate(s) hand hygiene action; all indications that apply at one moment must be recorded | | |
|  | **B Pt**: before touching a patient | | **A Pt**: after touching a patient |
|  | **B Asp**: before clean/aseptic procedure | | **A Sur**: after touching patient surroundings |
|  | **A Fl**: after body fluid exposure risk | | **A Pt fl**: after touching patient file |
| **HH action:** | response to the hand hygiene indication(s); it can be either a positive action by performing hand rub or handwash, or a negative action by missing hand rub or handwash. | | |
|  | Also, record appropriate use of gloves | | |
|  | **HR**: hand hygiene action by handrubbing with an alcohol-based formula  **HW**: hand hygiene action by handwashing with soap and water | | Missed: no hand hygiene action performed |

**
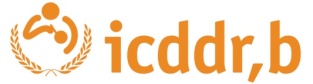
ANNEXURE-III: HOSPITAL PPE OBSERVATION FORM FOR HEALTHCARE WORKERS IN INDOOR UNIT**

Observation ID: _____________________ Date ___________ Start time: ____________

Department/outdoor ________________ Observer: _____________ End time: ___________

| **Mask type: 1=Surgical mask 2= KN95/equivalent 3= Cloth mask**  **4= N95 respirator 5= Other** |
| --- |

| SL | D/N/S  (d/n/s) | M/F  (M/F) | Mask  (Y/N) | Mask appro  (Y/N) | Mask wearing serial  (from outside to inside) | | | | Face shield | Caps | Gown | Trouser | Gum boot | Goggles | |
| --- | --- | --- | --- | --- | --- | --- | --- | --- | --- | --- | --- | --- | --- | --- | --- |
|  |  |  |  |  | m1 | m2 | m3 | m4 |  |  |  |  |  |  |  |
| hcw 1 |  |  |  |  |  |  |  |  |  |  |  |  |  |  |  |
| hcw 2 |  |  |  |  |  |  |  |  |  |  |  |  |  |  |  |
| hcw 3 |  |  |  |  |  |  |  |  |  |  |  |  |  |  |  |
| hcw 4 |  |  |  |  |  |  |  |  |  |  |  |  |  |  |  |
| hcw 5 |  |  |  |  |  |  |  |  |  |  |  |  |  |  |  |
| hcw 6 |  |  |  |  |  |  |  |  |  |  |  |  |  |  |  |
| hcw 7 |  |  |  |  |  |  |  |  |  |  |  |  |  |  |  |
| hcw 8 |  |  |  |  |  |  |  |  |  |  |  |  |  |  |  |
| hcw 9 |  |  |  |  |  |  |  |  |  |  |  |  |  |  |  |
| hcw 10 |  |  |  |  |  |  |  |  |  |  |  |  |  |  |  |
| hcw 11 |  |  |  |  |  |  |  |  |  |  |  |  |  |  |  |
| hcw 12 |  |  |  |  |  |  |  |  |  |  |  |  |  |  |  |

**Calculation:**

| Total Doctor Male: |  | Surgical |  |  |  |
| --- | --- | --- | --- | --- | --- |
| Total Doctor Female: |  | N95 |  |  |  |
| Total Nurse Male: |  | KN95 |  |  |  |
| Total Nurse Female: |  | Cloth |  |  |  |
| Total Cleaning staff Male: |  | Borkha |  |  |  |
| Total Cleaing staff Female: |  | N95+Surgical |  |  |  |
| Total No mask |  | KN95+Surgical |  |  |  |
| Total Mask wear: |  | Cloth+ Surgical |  |  |  |
| Total Appropriately wear: |  | Surgical+ Cloth |  |  |  |
| Total Face shield |  | Surgical+ Surgical |  |  |  |
| Total Caps |  | N95+Cloth |  |  |  |
| Total Gown |  | KN95+Cloth |  |  |  |
| Total Trouser |  |  |  |  |  |
| Total Gum boot |  |  |  |  |  |
| Total Goggles |  | **Total Appropriately wear:** | | | |

**
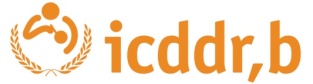
ANNEXURE-IV: HOSPITAL MASK-WEARING OBSERVATION IN OUTDOOR UNIT**

Observation ID: ________________________ Date ___________ Start time: ____________

Department/outdoor ________________ Observer: _____________ End time: ___________

| **Mask type: 1=Surgical mask 2= KN95/equivalent 3= Cloth mask**  **4= N95 respirator 5= Other** |
| --- |

| SL | M/F  (M/F) | Child  (C)* | Mask  (Y/N) | Mask appro  (Y/N) | Mask wearing serial  (from outside to inside) | | | | SL | M/F  (M/F) | Child  (C) | Mask  (Y/N) | Mask appro  (Y/N) | Mask wearing serial  (from outside to inside) | | | |
| --- | --- | --- | --- | --- | --- | --- | --- | --- | --- | --- | --- | --- | --- | --- | --- | --- | --- |
|  |  |  |  |  | m1 | m2 | m3 | m4 |  |  |  |  |  | m1 | m2 | m3 | m4 |
| 1 |  |  |  |  |  |  |  |  | 26 |  |  |  |  |  |  |  |  |
| 2 |  |  |  |  |  |  |  |  | 27 |  |  |  |  |  |  |  |  |
| 3 |  |  |  |  |  |  |  |  | 28 |  |  |  |  |  |  |  |  |
| 4 |  |  |  |  |  |  |  |  | 29 |  |  |  |  |  |  |  |  |
| 5 |  |  |  |  |  |  |  |  | 30 |  |  |  |  |  |  |  |  |
| 6 |  |  |  |  |  |  |  |  | 31 |  |  |  |  |  |  |  |  |
| 7 |  |  |  |  |  |  |  |  | 32 |  |  |  |  |  |  |  |  |
| 8 |  |  |  |  |  |  |  |  | 33 |  |  |  |  |  |  |  |  |
| 9 |  |  |  |  |  |  |  |  | 34 |  |  |  |  |  |  |  |  |
| 10 |  |  |  |  |  |  |  |  | 35 |  |  |  |  |  |  |  |  |
| 11 |  |  |  |  |  |  |  |  | 36 |  |  |  |  |  |  |  |  |
| 12 |  |  |  |  |  |  |  |  | 37 |  |  |  |  |  |  |  |  |
| 13 |  |  |  |  |  |  |  |  | 38 |  |  |  |  |  |  |  |  |
| 14 |  |  |  |  |  |  |  |  | 39 |  |  |  |  |  |  |  |  |
| 15 |  |  |  |  |  |  |  |  | 40 |  |  |  |  |  |  |  |  |
| 16 |  |  |  |  |  |  |  |  | 41 |  |  |  |  |  |  |  |  |
| 17 |  |  |  |  |  |  |  |  | 42 |  |  |  |  |  |  |  |  |
| 18 |  |  |  |  |  |  |  |  | 43 |  |  |  |  |  |  |  |  |
| 19 |  |  |  |  |  |  |  |  | 44 |  |  |  |  |  |  |  |  |
| 20 |  |  |  |  |  |  |  |  | 45 |  |  |  |  |  |  |  |  |
| 21 |  |  |  |  |  |  |  |  | 46 |  |  |  |  |  |  |  |  |
| 22 |  |  |  |  |  |  |  |  | 47 |  |  |  |  |  |  |  |  |
| 23 |  |  |  |  |  |  |  |  | 48 |  |  |  |  |  |  |  |  |
| 24 |  |  |  |  |  |  |  |  | 49 |  |  |  |  |  |  |  |  |
| 25 |  |  |  |  |  |  |  |  | 50 |  |  |  |  |  |  |  |  |

**Calculation:**

| Total male: |  | Surgical |  |  |  |
| --- | --- | --- | --- | --- | --- |
| Total female: |  | N95 |  |  |  |
| Total male-child: |  | KN95 |  |  |  |
| Total female-child: |  | Cloth |  |  |  |
| Total No Mask wear |  | Borkha |  |  |  |
| Total mask wear: |  | N95+Surgical |  |  |  |
| Appropriately wear: |  | KN95+Surgical |  |  |  |
| *Child=5-12 years based on observer assumption | | Cloth+ Surgical |  |  |  |
|  |  | Surgical+ Cloth |  |  |  |
|  |  | Surgical+ Surgical |  |  |  |
|  |  | N95+Cloth |  |  |  |
|  |  | KN95+Cloth |  |  |  |
|  |  | **Total Appropriately wear:** | | | |

**
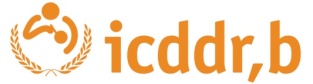
ANNEXURE-V: MONITORING CHECKLIST FOR IPC PRACTICES**

Hospital Name _____________________________________ID No: ___________________

Department_________________ Ward/Unit______________________ Date _______/_______/________

Observed by (Full name) _________________________________________________________________

Start time: ___________________________________End time: _____________________________________

Please visit each and every wad and filled up accordingly. Indicate with a tick **(√)** for **Yes** and Give **(X)** for **No** against the features observed. If no event occurs within the time period than mark with **(-),**

| **1. Hand hygiene** | **Doctor** | **Nurse** | **Patient** | **Attendant** | **Remarks** |
| --- | --- | --- | --- | --- | --- |
| 1.1 Hand wash basin/station/ facilities present |  |  |  |  |  |
| 1.2 No of working HW basin/station/ facilities present |  |  |  |  |  |
| 1.3 Running water present |  |  |  |  |  |
| 1.4 Soap/soapy water/detergent available |  |  |  |  |  |
| 1.5 Six step posters for hand wash available |  |  |  |  |  |
| 1.6 Tissue |  |  |  |  |  |
| 1.7 Towel |  |  |  |  |  |
| 1.8 Hand drier |  |  |  |  |  |
| 1.9 Hexisole / hand sanitizer available |  |  |  |  |  |
| 1.10 Hand station directly visible upon exiting patient zone |  |  |  |  |  |

| **2. Sharps management** | | |
| --- | --- | --- |
| 2.1 Sharp containers available for different kind of waste disposal with lid |  |  |
| 2.2 Needle crusher/ deforming hex present |  |  |
| **Process** | | |
| 2.3 All sharps are kept in protected safety and sealed jar/ container |  |  |
| 2.4 Needles are destroyed/ deformed before keeping in jar/ container |  |  |
| 2.5 All sharps are collected with highest care/ precaution using utility gloves |  |  |

| **3. Spill management** | | |
| --- | --- | --- |
| 3.1 Spill covering gauze/ towels present |  |  |
| 3.2 Readily accessible with spill covering gauze/ towel reserving container |  |  |
| 3.3 Designated drum / container for keeping soiled clothes |  |  |
| 3.4 Body fluid spills are covered as soon as possible (including vomit/ urine/ stool/cough/spit) |  |  |
| 3.5 Soiled gauze/ towels are kept in a particular designated drum/container |  |  |

| **4. Waste management** | | |
| --- | --- | --- |
| 4.1 Required color bins with closed lids present at workplace |  |  |
| 4.2 3 bins (Red/ Yellow/ Black) present for final disposal within hospital area |  |  |
| 4.3 PPE available for waste management |  |  |
| **Process** | | |
| 4.4 Wastes are segregated at workplace |  |  |
| 4.5 Infectious sharps waste are kept in puncture proof container |  |  |
| 4.6 Waste bins are filled no more than 2/3 full |  |  |
| 4.7 Sharps bins are filled no more than 2/3 full |  |  |

| **5. PPE (Personal protective equipment)** | | |
| --- | --- | --- |
| 5.1 Separate Donning Doffing area |  |  |
| 5.2 Specific color-coded bins/biohazard bags for the disposal of PPE |  |  |
| 5.3 Nurses maintaining hand hygiene during distribution of medicine |  |  |
| 5.4 Cleaning staff maintaining hand hygiene during distribution of food |  |  |

| **6. Miscellaneous** | |
| --- | --- |
| 6.1 Number of beds in the ward |  |
| 6.2 Number of patients in that ward |  |
| 6.3 Number of attendants |  |
| 6.4 Number of patients wearing mask |  |
| 6.5 Number of attendant/visitors wearing face shield |  |
| 6.6 Number of attendant /visitors wearing mask |  |
| 6.7 Number of children (<10year) as attendant/visitor |  |
| 6.8 Number of attendant/visitors having hand sanitizer |  |

**ANNEXURE-VI: IPC KAP SURVEY QUESTIONNAIRE FOR HEALTHCARE PROVIDERS**

Name (নাম): ___________________________________Survey ID (for icddrb)____________________

Mobile Number (মোবাইল নাম্বার):____________ _________ Date (তারিখ) ____/______/______________

| **1** | **Demographic information** | **Code** | |
| --- | --- | --- | --- |
| 1.1 | Age (In completed years): বয়স ( বছর ) |  | |
| 1.2 | Sex (লিঙ্গ) | 0=Female (মহিলা )  1=Male (পুরুষ ) | |
| 1.3 | Education (শিক্ষাগত যোগ্যতা) | 1=MBBS/Honors and above  2=BSC/Diploma in nursing  3=Diploma in MT  4=Other ……………….. | 5=No education  6=class 1-Class 5  7= Class 6-Class 10  8= Class 11-Class 12 |
| 1.4 | Present designation (বর্তমান পদবী) | 1= Physician 2= Nurse 3= Medical Technologist  4= Cleaning Staff 5= Administrative Staff | |
| 1.5 | Present Institution (বর্তমান প্রতিষ্ঠান) |  | |
| 1.6 | How long have you been in this profession? আপনি এই পেশায় কত দিন ধরে আছেন? | ……………..Year…………………..Month……………… | |
| 1.7 | Present working unit  (বর্তমান কর্মরত ইউনিট) | 1=Medicine/Gastro/Transfusion/Ortho  2=Pediatrics/Dialysis  3=Obs/Gyne  4=Surgery /Psycho  5=Other _____________________ | |
| 1.8 | How long you have been in this unit  (এই ইউনিটে কত দিন যাবত কাজ করছেন) | ……………..Year…………………..Month……………… | |

| **2** | | **Knowledge** | | |  | | |
| --- | --- | --- | --- | --- | --- | --- | --- |
| 2.1 | | I am familiar with hospital infection prevention and control (IPC) manuals and guidelines  (হাসপাতালে জীবাণু সংক্রমণ নিয়ন্ত্রণ/ ইনফেকশন কন্ট্রোল ম্যানুয়াল এর নির্দেশনাগুলির সাথে আমি পরিচিত) | | | 1=Agree (সম্মত)  2=Disagree (অসম্মত) | | 3=Don’t know (জানিনা)  4=N/A (প্রযোজ্য নয় ) |
| 2.2 | | I know the World Health Organization’s (WHO) 5 moments of hand hygiene (আমি বিশ্ব স্বাস্থ্য সংস্থার হাত পরিষ্কারের পাঁচটি মুহূর্ত্ব সম্পর্কে জানি) | | | 1=Agree (সম্মত)  2=Disagree (অসম্মত) | | 3=Don’t know (জানিনা)  4=N/A (প্রযোজ্য নয় ) |
| 2.2.1 | | World Health Organization’s 5 moments of hand hygiene are –  ( বিশ্ব স্বাস্থ্য সংস্থার হাত পরিষ্কারের পাঁচটি মুহূর্তগুলো হলো - )  ( Please tick the point that mentioned, multiple-choice answers) | | | 1=Before touching the patient  (রোগীকে স্পর্শ করা পূর্বে)  2=After touching the patient  (রোগীকে স্পর্শ করা পর)  3=Before conducting any aseptic procedure  (যে কোনো নির্বীজ কাজ করার পূর্বে)  4=After risk of potential body fluid exposure  (রোগীর শরীর থেকে তরল নিঃসৃত হবার ঝুঁকি থাকলে)  5= After touching patient’s surroundings  (রোগীর পারিপার্শ্বিক পরিবেশ স্পর্শ করার পর) | | |
| 2.3 | | Nosocomial infection is an infection that the patient brings from home (নসোকোমিয়াল ইনফেকশন এমন একটি সংক্রমণ যা রোগী বাসা থেকে নিয়ে আসে) | | | 1=Agree (সম্মত)  2=Disagree (অসম্মত) | | 3=Don’t know (জানিনা)  4=N/A (প্রযোজ্য নয় ) |
| 2.4 | | Standard precautions apply to all patients regardless of their diagnosis (রোগীর যে রোগ নির্ণয় হোক না কেন, স্ট্যান্ডার্ড সতর্কতাগুলি সবার জন্য প্রযোজ্য) | | | 1=Agree (সম্মত)  2=Disagree (অসম্মত) | | 3=Don’t know (জানিনা)  4=N/A (প্রযোজ্য নয় ) |
| 2.5 | | I am familiar with isolation and cohorting of infectious patients  (আমি সংক্রামক রোগীদের আইসোলেশন ও কোহোর্টিং সাথে পরিচিত) | | | 1=Agree (সম্মত)  2=Disagree (অসম্মত) | | 3=Don’t know (জানিনা)  4=N/A (প্রযোজ্য নয় ) |
| 2.5.1 | | I should go through isolation and cohorting of patients when –  ( আপনি কখন রোগীদের জন্যে আইসোলেশন এবং কোহোর্টিং ব্যবহার করবেন ? ) | | | 1=Patients have signs/symptoms of infection  (রোগীদের ইনফেকশন এর লক্ষণ/উপসর্গ আছে)  2=The hospital does not have sufficient rooms for isolation and hence should go for cohorting  (হাসপাতালে আইসোলেশন এর জন্যে যথেষ্ট সংখ্যক কক্ষ নেই সেই জন্যে কোহোর্টিং করতে হবে)  3= Others [Specify] | | |
| 2.6 | | Washing hands with soap or hand sanitizer decreases the risk of transmission of infections  (সাবান বা হ্যান্ড স্যানিটাইজার দিয়ে হাত পরিষ্কার করলে জীবাণু সংক্রমণের সম্ভাবনা কমে ) | | | 1=Agree (সম্মত)  2=Disagree (অসম্মত) | | 3=Don’t know (জানিনা)  4=N/A (প্রযোজ্য নয় ) |
| 2.7 | | Use of a hand sanitizer for hand hygiene is as effective as soap and water if hands are not visibly dirty  (হাতে দৃশ্যমান ময়লা না থাকলে হাত পরিষ্কারের জন্যে হ্যান্ড স্যানিটাইজার বা সাবান-পানি উভয়ই সমান কার্যকরী ) | | | 1=Agree (সম্মত)  2=Disagree (অসম্মত) | | 3=Don’t know (জানিনা)  4=N/A (প্রযোজ্য নয় ) |
| 2.8 | | There is no need to wash hands prior to patient contact, if hands are not visibly dirty  (হাতে দৃশ্যমান ময়লা না থাকলে রোগীর সংস্পর্শে আসার পূর্বে বা পরে হাত ধোয়ার প্রয়োজন নেই ) | | | 1=Agree (সম্মত)  2=Disagree (অসম্মত) | | 3=Don’t know (জানিনা)  4=N/A (প্রযোজ্য নয় ) |
| 2.9 | | Gloves provide complete protection against acquiring or transmitting infection  (জীবাণু দ্বারা আক্রান্ত হওয়া এবং জীবাণু ছড়ানোর ক্ষেত্রে গ্লাভস পূর্ণ নিরাপত্তা দেয় ) | | | 1=Agree (সম্মত)  2=Disagree (অসম্মত) | | 3=Don’t know (জানিনা)  4=N/A (প্রযোজ্য নয় ) |
| 2.10 | | I can wear the same pair of gloves for multiple patients as long as there is no visible contamination  (গ্লাভসে দৃশ্যমান ময়লা না থাকলে আমি একাধিক রোগীর জন্যে একই গ্লাভস পরিধান করতে পারবো ) | | | 1=Agree (সম্মত)  2=Disagree (অসম্মত) | | 3=Don’t know (জানিনা)  4=N/A (প্রযোজ্য নয় ) |
| 2.11 | | All medical equipment equipment requires disinfection after use  (সমস্ত চিকিৎসা সরঞ্জাম ব্যবহারের আগে নির্বীজন/ স্টেরিলাইজেশন করা উচিৎ ) | | | 1=Agree (সম্মত)  2=Disagree (অসম্মত) | | 3=Don’t know (জানিনা)  4=N/A (প্রযোজ্য নয় ) |
| 2.12 | | High-touch surfaces should be cleaned more frequently than low-touch surfaces to reduce hospital aquired infections  (হাসপাতালে জীবাণু সংক্রমণ কমাতে লো-টাচ স্থানগুলির তুলনায় হাই--টাচ স্থানগুলি ঘন ঘন পরিষ্কার করা উচিত ) | | | 1=Agree (সম্মত)  2=Disagree (অসম্মত) | | 3=Don’t know (জানিনা)  4=N/A (প্রযোজ্য নয় ) |
| 2.13 | | Medical waste bins must be filled no more than 2/3^rd^ full  ( মেডিকেল বর্জ্য বিন 2/3 এর বেশি পূর্ণ করা উচিত নয় ) | | | 1=Agree (সম্মত)  2=Disagree (অসম্মত) | | 3=Don’t know (জানিনা)  4=N/A (প্রযোজ্য নয় ) |
| 2.14 | | Isolation and cohorting are only needed in outbreak situations  (আইসোলেশন ও কোহোর্টিং শুধুমাত্র রোগের প্রাদুর্ভাবে প্রয়োজন) | | | 1=Agree (সম্মত)  2=Disagree (অসম্মত )  3=Don’t know (জানিনা)  4=N/A (প্রযোজ্য নয়) | |  |
| 2.15 | | It is compulsory for hospitals to have a dedicated IPC committee for infection control  (সংক্রমণ নিয়ন্ত্রণের জন্য হাসপাতালে একটি নির্দিষ্ট আইপিসি কমিটি থাকা বাধ্যতামূলক ) | | | 1=Agree (সম্মত)  2=Disagree (অসম্মত) | | 3=Don’t know (জানিনা)  4=N/A (প্রযোজ্য নয় ) |
| **3** | **Attitude** | |  | | | |  |
| 3.1 | Policies and procedures for infection control should be adhered to at all the times (সংক্রমণ নিয়ন্ত্রণের নীতিমালা এবং পদ্ধতিগুলি সর্বদা মেনে চলা উচিত) | | 1=Agree (সম্মত)  2=Disagree (অসম্মত) | | | 3=Don’t know (জানিনা)  4=N/A (প্রযোজ্য নয় ) |  |
| 3.2 | I feel that the infection control policies and guidelines are sufficient in the hospital (আমি মনে করি, সংক্রমণ নিয়ন্ত্রণের নীতিমালা এবং গাইডলাইন এই হাসপাতালের সংক্রমণ নিয়ন্ত্রণে যথেষ্ট) | | 1=Agree (সম্মত)  2=Disagree (অসম্মত) | | | 3=Don’t know (জানিনা)  4=N/A (প্রযোজ্য নয় ) |  |
| 3.3 | I have enough time to comply with infection prevention guidelines (সংক্রমণ নিয়ন্ত্রণের গাইড লাইন মেনে চলার জন্য আমার পর্যাপ্ত সময় রয়েছে) | | 1=Agree (সম্মত)  2=Disagree (অসম্মত) | | | 3=Don’t know (জানিনা)  4=N/A (প্রযোজ্য নয় ) |  |
| 3.4 | Resources are available to comply with infection prevention guidelines in this hospital (সংক্রমণ নিয়ন্ত্রণের গাইড লাইন মেনে চলার জন্য অত্র হাসপাতালে পর্যাপ্ত উপকরণ/সম্পদ রয়েছে) | | 1=Agree (সম্মত)  2=Disagree (অসম্মত) | | | 3=Don’t know (জানিনা)  4=N/A (প্রযোজ্য নয় ) |  |
| 3.5 | Patients expect me to wash hands before & after touching them  (রোগীরা আশা করেন যে - তাদের স্পর্শ করার আগে ও পরে আমি যেন হাত ধুয়ে নেই ) | | 1=Agree (সম্মত)  2=Disagree (অসম্মত) | | | 3=Don’t know (জানিনা)  4=N/A (প্রযোজ্য নয় ) |  |
| 3.6 | The workload affects my ability to follow infection prevention guidelines (কাজের চাপের কারণে আমার সংক্রমণ নিয়ন্ত্রণের গাইড লাইন মেনে চলা ব্যহত হয়) | | 1=Agree (সম্মত)  2=Disagree (অসম্মত) | | | 3=Don’t know (জানিনা)  4=N/A (প্রযোজ্য নয় ) |  |
| 3.7 | Overcrowded working area increases transmission of Infection **(**অতিরিক্ত ভীড় সম্বলিত কর্মক্ষেত্র জীবাণু সংক্রমণের ঝুঁকি বাড়িয়ে দেয় **)** | | 1=Agree (সম্মত)  2=Disagree (অসম্মত) | | | 3=Don’t know (জানিনা)  4=N/A (প্রযোজ্য নয় ) |  |
| 3.8 | Following standard operation procedures decreases the risk of contamination স্ট্যান্ডার্ড অপারেশন পদ্ধতি অনুসরণ করলে সংক্রমণের ঝুঁকি কমে যায় | | 1=Agree (সম্মত)  2=Disagree (অসম্মত) | | | 3=Don’t know (জানিনা)  4=N/A (প্রযোজ্য নয় ) |  |
| 3.9 | Practicing adequate personal hygiene decreases the risk of contamination **(**পর্যাপ্ত ব্যাক্তিগত পরিচ্ছন্নতা বজায় রাখলে সংক্রমণের ঝুঁকি কমে যায় **)** | | 1=Agree (সম্মত)  2=Disagree (অসম্মত) | | | 3=Don’t know (জানিনা)  4=N/A (প্রযোজ্য নয় ) |  |
| 3.1110 | Used sharps **must** be discarded in puncture-proof containers **(**ব্যবহৃত ধারালো জিনিসপত্র পাঙ্কচার নিরোধকারী কন্টেইনারে ফেলা উচিত **)** | | 1=Agree (সম্মত)  2=Disagree (অসম্মত) | | | 3=Don’t know (জানিনা)  4=N/A (প্রযোজ্য নয় ) |  |
| 3.1211 | Needle stick or sharp injuries should be immediately documented and reported to the higher authority **(**সুঁই বা যে কোনো ধারালো জিনিসপত্রের মাধ্যমে আঘাতপ্রাপ্ত হলে সঙ্গে সঙ্গে তা লিপিবদ্ধ করা ও উর্ধ্বতন কর্তৃপক্ষকে জানানো উচিত**)** | | 1=Agree (সম্মত)  2=Disagree (অসম্মত) | | | 3=Don’t know (জানিনা)  4=N/A (প্রযোজ্য নয় ) |  |
| 3.1312 | Wound dressing can be disposed of in an ordinary garbage bin  **(**ক্ষত এর ড্রেসিং এর গজতুলা সাধারণ ময়লার বিনে ফেলা যায়**)** | | 1=Agree (সম্মত)  2=Disagree (অসম্মত) | | | 3=Don’t know (জানিনা)  4=N/A (প্রযোজ্য নয় ) |  |
| 3.1413 | Decontaminating equipment with 10% sodium hypochlorite for 10 minutes is enough **(**যন্ত্রপাতিকে ১০% সোডিয়াম হাইপোক্লোরাইট দিয়ে ১০ মিনিট জীবাণুমুক্ত করাই যথেষ্ট **)** | | 1=Agree (সম্মত)  2=Disagree (অসম্মত) | | | 3=Don’t know (জানিনা)  4=N/A (প্রযোজ্য নয় ) |  |
| **4** | **Practice** | |  | | | |  |
| 4.1 | I wash hands before and after direct contact with the patient (রোগীর সংস্পর্শে আসার আগে এবং পরে আমি হাত ধুয়ে থাকি) | | 1=Always (সব সময়)  2=Often (প্রায় সময়)  3=Sometimes (কখনো কখনো) | | | 4= Seldom (কদাচিৎ)  5=Never (কখনো না )  6=N/A(প্রযোজ্য নয় ) |  |
| 4.2 | I wash hands before and after wearing gloves  (গ্লাভস পরার আগে এবং পরে আমি হাত ধুয়ে থাকি) | | 1=Always (সব সময়)  2=Often (প্রায় সময়)  3=Sometimes (কখনো কখনো) | | | 4= Seldom (কদাচিৎ)  5=Never (কখনো না )  6=N/A(প্রযোজ্য নয় ) |  |
| 4.3 | I wear gloves when performing invasive procedures (cannula, catheter etc) ইনভেসিভ পদ্ধতিগুলো সম্পাদন করার সময় আমি গ্লাভস পরে থাকি(ক্যানোলা, ক্যাথেটার ইত্যাদি) | | 1=Always (সব সময়)  2=Often (প্রায় সময়)  3=Sometimes (কখনো কখনো) | | | 4= Seldom (কদাচিৎ)  5=Never (কখনো না )  6=N/A(প্রযোজ্য নয় ) |  |
| 4.4 | I attend in-service training/workshops related to infection control regularly (আমি নিয়মিত সংক্রমণ নিয়ন্ত্রণ সম্পর্কিত ট্রেনিং/ওয়ার্কশপ গুলোতে অংশ নিয়ে থাকি) | | 1=Always (সব সময়)  2=Often (প্রায় সময়)  3=Sometimes (কখনো কখনো) | | | 4= Seldom (কদাচিৎ)  5=Never (কখনো না )  6=N/A(প্রযোজ্য নয় ) |  |
| 4.5 | I dispose of sharp instruments immediately after use in a puncture-proof container  ধারালো যন্ত্রপাতি গুলো **(**সূই/নিডল) ব্যবহারের সাথে সাথে আমি নষ্ট করে ফেলি | | 1=Always (সব সময়)  2=Often (প্রায় সময়)  3=Sometimes (কখনো কখনো) | | | 4= Seldom (কদাচিৎ)  5=Never (কখনো না )  6=N/A(প্রযোজ্য নয় ) |  |
| 4.6 | I dispose used gloves & other items in the proper place  ব্যবহৃত গ্লোভস এবং অন্যান্য জিনিসপত্র গুলো আমি যথাযথ স্থানে রাখি বা ডিসপোজ করি | | 1=Always (সব সময়)  2=Often (প্রায় সময়)  3=Sometimes (কখনো কখনো) | | | 4= Seldom (কদাচিৎ)  5=Never (কখনো না )  6=N/A(প্রযোজ্য নয় ) |  |
| 4.7 | I change gloves between patients irrespective of a patient’s infectious status (প্রতিটি রোগীর সংক্রামক অবস্থা বিবেচনা করে আমি গ্লাভস পরিবর্তন করি) | | 1=Always (সব সময়)  2=Often (প্রায় সময়)  3=Sometimes (কখনো কখনো) | | | 4= Seldom (কদাচিৎ)  5=Never (কখনো না )  6=N/A(প্রযোজ্য নয় ) |  |
| 4.8 | I wear a gown/apron if soiling with blood or body fluids is likely (রক্ত বা তরল জাতীয় পদার্থ ছড়ানোর সম্ভাবনা থাকলে আমি গাউন / এপ্রোন পরে থাকি) | | 1=Always (সব সময়)  2=Often (প্রায় সময়)  3=Sometimes (কখনো কখনো) | | | 4= Seldom (কদাচিৎ)  5=Never (কখনো না )  6=N/A(প্রযোজ্য নয় ) |  |
| 4.9 | I wear a disposable facemask whenever there is a possibility of a splash or splatter (কিছু ছি্টকে/ছিটে আসার সম্ভাবনা থাকলে আমি ডিস্পোজেবল ফেস মাস্ক পরে থাকি) | | 1=Always (সব সময়)  2=Often (প্রায় সময়)  3=Sometimes (কখনো কখনো) | | | 4= Seldom (কদাচিৎ)  5=Never (কখনো না )  6=N/A(প্রযোজ্য নয় ) |  |
| 4.10 | Staff/cleaners clean up blood spills immediately using disinfectant কর্মীরা জীবাণুনাশক ব্যবহার করে ছড়িয়ে পড়া রক্ত সাথে সাথে পরিষ্কার করে ফেলে | | 1=Always (সব সময়)  2=Often (প্রায় সময়)  3=Sometimes (কখনো কখনো) | | | 4= Seldom (কদাচিৎ)  5=Never (কখনো না )  6=N/A(প্রযোজ্য নয় ) |  |
| 4.11 | Do you use hand sanitizer? (hexisol/hand rub etc)  আপনি কি হ্যান্ড-স্যানিটাইজার ব্যবহার করেন? | | 1=Always (সব সময়)  2=Often (প্রায় সময়)  3=Sometimes (কখনো কখনো) | | | 4= Seldom (কদাচিৎ)  5=Never (কখনো না )  6=N/A(প্রযোজ্য নয় ) |  |
| 4.12 | I inform other healthcare personnel about suspected infectious patients before transfer of the patients  (আমি সংক্রামক রোগীদের স্থানান্তর করার আগে তাদের সম্পর্কে অন্যান্য স্বাস্থ্যকর্মীদের এ রোগ সম্পর্কে অবগত করি) | | 1=Yes ( হ্যাঁ )  2=No (না )  3=N/A(প্রযোজ্য নয় ) | | |  |  |
| 4.13 | I inform authorities about patients with highly transmissible infections (e.g. Corona virus, measles, Tuberculosis, Nipah etc.)  আমি অতি সংক্রামক রোগ এ (যেমন-করোনার ভাইরাস, হাম, যক্ষা, নিপাহ ভাইরাস ইত্যাদি) আক্রান্ত রোগীদের সম্পর্কে কর্তৃপক্ষকে অবহিত করি | | 1=Yes ( হ্যাঁ )  2=No (না )  3=N/A (প্রযোজ্য নয় ) | | |  |  |
| 4.14 | What are the barriers to using hand sanitizers? (হ্যান্ড-স্যানিটাইজার ব্যবহারে বাধা কী কী?) | 1= Costly  2= Insufficient supply  3= Skin reaction  4= Shortage of time  5= Lack of awareness | | | 6= Apathy  7= Lack of motivation  8= Lack of facility  9= Lack of interest  10= No barrier  11=Other___________________ | |  |
| 4.15 | What are the barriers to performing hand washing? **(**হাত ধোয়ার ক্ষেত্রে বাধাগুলি কী কী?) | 1=Insufficient supply to washing agent  2= Quality of washing reagent  3= Workload Shortage of time  4= Lake of awareness/knowledge  5= Laziness | | | 6= Lack of motivation  7= Lack of facility (runny water /basin)  8= Forgetfulness  9= No barrier  10=Other___________________ | |  |

Data collected and checked by: **.................................... Date……………………………**

**ANNEXURE-VI
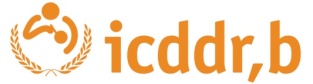
I: PHYSICIAN ANTIBIOTIC PRACTICES SURVEY (ENGLISH)**

Survey ID: ____________ Date: ____/______/______________ Hospital Name: ______Department: _______

Physician Name:_________ Mobile Number:__________ Designation____ Graduation year (MBBS):___

|  | **Question** | | **Category** | |
| --- | --- | --- | --- | --- |
| 1 | Antimicrobial resistance (AMR) is a significant problem **(Tick all that apply)** | | - 1=Worldwide 2=In my country - 3=In my hospital4=In my daily practice - 5=It is not a problem in my setting | |
| 2 | According to you, which of the followings is true about antimicrobial resistance (AMR)? **(Tick all that apply)** | | - 1=A patient is highly likely to develop drug-resistance during their hospital stay - 2=Very high proportion (>30%) of gram-negative infections are highly drug-resistant - 3=A high proportion (>30%) of Staphylococcal infections are resistant to methicillin | |
| 3 | Which of the following are causes of AMR in your setting? **(Tick all that apply)** | | - 1=Inappropriate use of antibiotics - 2=Easy access to antibiotics without a prescription - 3=Over prescription of broad-spectrum antibiotics - 4=Lack of adequate diagnostic tests, which leads to overuse of antibiotics - 5=Sporadic supply of antibiotics, which leads to interruptions of therapy - 6=Lack of close clinical follow-up during antibiotic treatment - 7=Patient demands and expectations about receiving antibiotics - 8=Poor infection control practices by health professionals - 9=Antibiotics available in the hospital are of poor quality | |
| 4 | Which of the following represent your approach to prescribing antibiotics? **(Tick all that apply)** | | - 1=I routinely step down IV antibiotics to PO antibiotics as soon as possible - 2=I routinely narrow the spectrum of antibiotics chosen - 3=Cost considerations for the patient affects my choice of antibiotics - 4=I routinely choose very broad-spectrum antibiotics empirically because most patients are infected with drug-resistant organisms - 5=I routinely choose very broad-spectrum antibiotics empirically because microbiology results are not available in a timely fashion - 6=I routinely check microbiology laboratory results to guide my choice of antibiotics | |
| 5 | Do you know about Antimicrobial Stewardship Programs? | | - 1=Yes 2=No | |
| 6 | I believe the following regarding Antimicrobial Stewardship Programs (ASP) **(Tick all that apply)** | | - 1=The hospital should have the capacity to establish and implement an effective ASP - 2=I would like more feedback on my antibiotic selections - 3=ASP can be an obstacle to good patient care - 4=ASP overrides prescribers’ decision autonomy - 5=Infectious diseases experts should be available in my hospital - 6=Pharmacists with sufficient training to provide guidance on antibiotic usage should be available in my hospital | |
| 7* | Which ASP interventions would be most successful at your hospital  **Arrange in order**  **1= Most helpful**  **6= Lowest helpful** | | - ____Education on antimicrobial therapy to medical staff - ____ Develop new institutional guidelines for empiric antimicrobial use - ____ Access to institution-specific antibiogram for treating teams - ____ Implementation of antibiotic audit and feedback - ____ Antibiotic restriction guideline - ____ Improved access to timely diagnostics | |
| 8 | When a healthy and stable adult patient presents with fever and pain during urination, which of the following should you do? **(Tick only one)** | | - 1=No need for urine testing - 2=Send urine for urinalysis/ urine R/E - 3=Send urine for urine culture - 4= Send for culture only if urinalysis or urine R/E positive - 5= Send for both Urine R/E and C/S | |
| 9 | In regards to question #**8**, is that approach always feasible? | | Yes No If no, what are the barriers?  1_____________________________________________________  2_____________________________________________________ | |
| 10 | For the patient above, what should you do about starting antibiotic treatment? **(Tick one)** | | - 1=Start antibiotic without sending a urine culture - 2=Start antibiotic while urinalysis and C/S is pending - 3= Start antibiotic after receiving urine R/E results - 4=Wait for a positive culture before starting antibiotics | |
| 11 | In regards to question **#10,** is that approach always feasible?**(Tick one)** | | Yes No If no, what are the barriers?  1_____________________________________________________  2_____________________________________________________ | |
| 12 | What is your first line antibiotic for uncomplicated UTI (OPD)?**(Tick one)** | | - 1=Ceftriaxone 2=Ciprofloxacin or other FQ - 3= Trimethoprim/Sulfamethoxazole 4=Nitrofurantoin - 5=Other(please specify) ___________________________ | |
| 13 | What is your first line antibiotic treatment for a complicated UTI (IPD)?**(Tick one)** | | - 1=Ceftriaxone 2=Ciprofloxacin or another fluoroquinolone - 3=Carbapenem 4= Piperacillin-Tazobactam - 5= Other(please specify) ___________________________ | |
| 14 | Where did you learn this approach? | |  | |
| **[Please complete the next section if you are involved in the care of surgical/post-operative patients]** | | | | |
| 15 | | What type of surgical patients do you most commonly manage?  **(Tick one)** | | - 1=OBS &GYN 2=General Surgery - 3=Genitourinary 4=Cardiac/Thoracic/Vascular - 5=Orthopedic 6=Other(Plz Specify)_____________ |
| 16 | | Do you routinely prescribe Surgical Antibiotic Prophylaxis (SAP) for clean-contaminated procedures (defined as operative wounds in which a viscus is entered under controlled conditions and without unusual contamination?) | | - 1=Yes - 2=No |
| 17 | | If you answered yes above, what is the antibiotic you most commonly prescribe for SAP?  **(Tick one)** | | - 1=Cefazolin2=Ceftriaxone - 3=Vancomycin4=Ciprofloxacin or another fluoroquinolone - 5=Trimethoprim/Sulfamethoxazole6=Gentamycin - 7=Other(Plz specify)______________________________ |
| 18 | | What is your usual duration for SAP in a clean-contaminated case, without signs of infection?  **(Tick one)** | | - 1=One dose only (within 30-60 mins of surgery) 2=More than one dose, but < 24 hours - 3=1-3 days 4=3-7 days - 5=More than 7 days |
| 19 | | If you answered 3-5 above (Question **No. 18**); do you think it is safe and feasible to discontinue SAP at 24 hours post-surgery in your setting? | | - 1=Yes - 2=No |
| 20 | | Where did you learn your approach to surgical antibiotic prophylaxis? | |  |

**Data collected and checked by:__________**

**
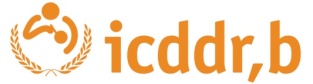
ANNEXURE-VIII: FOCUS GROUP DISCUSSION (FGD) GUIDELINE WITH HEALTHCARE PROVIDERS-IPC (ENGLISH)**

**Protocol Title:** Strengthening the Function of Infection Prevention & Control and Antimicrobial Stewardship Committees in Tertiary Care Hospitals in Bangladesh **Protocol No. PR-**20027

**Introduction:** Today we will discuss infection prevention and control in your hospital setting and how can we improve the practices

- 1. **Overview and ideas about IPC**
  2. Why is infection prevention & control (IPC) important? Why do we need to improve IPC practices? What initiative will you take to improve IPC practices?
  3. What initiative should the hospital authorities take to improve the IPC situation?
  4. Discuss the existing IPC committees in this hospital. Who should be included in the committee? How should they be made more functional?
  5. Why are healthcare service providers (HSPs) at risk of HAI?
  6. Discuss how we can protect HSPs through IPC (handwashing, waste disposal, isolation, cleanliness & disinfections, education and training, etc).
  7. Discuss using hand rub versus handwashing? (availability, cost, practices).
  8. **Vaccine-preventable diseases for healthcare services providers**
     - - 1. What are healthcare-associated diseases that are vaccine-preventable?
         2. What do you know about (I) Hepatitis B Virus and vaccine (II) Influenza & Vaccine (Transmission routes, immunization, Prevention measures, safety, screening, cost, who should provide)?
  9. **Isolation and cohorting**
     - - 1. Discuss common diseases (commonly occurring in our country) that require Isolation and Cohorting (signs and symptoms, transmission route, protection).
         2. How frequently did you encounter such cases (last year)? What barriers did you face to isolate such cases?
         3. Have you or your colleagues seen any patient who need to be isolated but you did not? Why (reporting person)?
  10. **Challenges regarding implementation of infection prevention and control.**

1. What are the key challenges you face while implementing any IPC? How did you deal with the situation? Would you please share some examples?
2. Would you please share your opinion on whether those were handled appropriately or otherwise?
3. What would you recommend to improve IPC activities?
4. What can be done within the existing resources?
   1. **Are you aware of any IPC guidelines/policies and implementation of policies?**
   2. If yes, what are they?
   3. Do you have any IPC guidelines at your hospital? If yes, please share the different components of the hospital IPC policy.
   4. If not, do you think we should have a hospital-level IPC policy? Why and why not? What components should be in the IPC guideline?
   5. **Availability of personal protective equipment**
   6. How frequently do you use personal protective equipment (PPE) to protect against cross-infection? (availability, barrier, practice)
   7. Briefly discuss COVID IPC measures

**
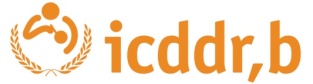
ANNEXURE-IX: FOCUS GROUP DISCUSSION(FGD) GUIDELINE WITH PHYSICIANS REGARDING ANTIBIOTIC USE (ENGLISH)**

**Introduction:** Today we will discuss AMR and rational use of antibiotic use at your hospital and how can we improve the practices

**Questions for Focus Group Discussion**

1. How often do you consider AMR when prescribing antibiotics?
2. The point of this is to think about their prescribing practices driving the problem
3. Do you get adequate teaching on MDROs/AMR/antibiotic prescribing best practices? What do you think is missing or could be improved?
   - - - 1. Are providers familiar with antibiotic prescribing guidelines? Or do they continue practices just because “this is how it is done”? Which level of doctors makes antibiotic prescribing decisions?
         2. While they know what the international textbooks say, how comfortable are they in practising this in the Bangladesh setting? If not comfortable, why? What would it take to be more comfortable? i.e. Local studies, Senior docs/Professors who change their practices first, National Guidelines? List in order
         3. What are the main reasons they do not follow up-to-date practices in prescribing antibiotics including choice of antibiotics and duration of treatment?
         4. Who do they think should lead the effort for rational prescribing of antibiotics in their hospital? The doctors, the administrators? The nurses? Only the Professors at the Medical College?
         5. What does evidence-based medicine mean to you? How is evidence-based medicine practiced in your hospital?
4. Does the government need to teach the public about the high rates of antibiotic resistance so that the public will be more knowledgeable? Do you think it will change patient expectations around antibiotic use? If so, what do you think would be the best/most effective way to impart this message (schools, TV, etc.)?
5. Do you believe that your hospital can establish and implement an effective ASP? How do you foresee that working? Do you foresee any issues and barriers to any specific approaches? What would be the best way to implement an ASP program in your hospital?
